# Long‐term evolution of the structure of the St. Lawrence (Canada) marine ecosystem in the context of climate change and anthropogenic activities: An isotopic perceptive

**DOI:** 10.1002/ece3.10740

**Published:** 2023-11-28

**Authors:** Ève Rioux, Jory Cabrol, Véronique Lesage

**Affiliations:** ^1^ Fisheries and Oceans Canada Maurice Lamontagne Institute Mont‐Joli Québec Canada

**Keywords:** community modification, environmental change, isotopic shift, resource partitioning, trophic niche, trophodynamics

## Abstract

Documenting long‐term changes in the trophic structure of food webs and how species respond to these changes is essential to forecast their vulnerability and resilience to environmental stressors. Over the past decades, the St. Lawrence marine ecosystem (Canada) has experienced major changes in its physical, chemical, and biological conditions from overfishing, acoustic and chemical pollution, climate change, and the increased abundance of some top predators. These changes have likely affected the trophodynamics of the ecosystem, and are suspected to have deleterious effects on endangered species of mammals and other components of the ecosystem, such as blue whales (*Balaenoptera musculus*), fin whales (*B. physalus*), and beluga (*Delphinapterus leucas*). This study examined the trophic structure of the St. Lawrence marine ecosystem, including the isotopic niche of various species, over two periods of contrasting pressures from anthropogenic and climatic stressors (1995–2003 vs. 2019–2021). Stable isotope ratios were measured in 1240 samples of 21 species of marine invertebrates, fishes, and mammals sampled during both periods. A significant change in the isotopic value and niche position between periods is observed in most of the sampled species. While the direction of change and effect size were not uniform among species, these changes confirmed that substantial modifications in community structure have occurred over time. Niche overlap decreased considerably among some of the pelagic and demersal fishes, and among whale species during the most recent period. Combined with a concomitant reduction in niche breadth in several species, these observations suggested that resource sharing was limited among these species. This study highlighted some degree of dietary plasticity in several species, and a long‐term change in the trophic structure of the St. Lawrence marine ecosystem, with likely effects on diet composition and energetics of several populations, including endangered species.

## Graphical abstract

Bayesian standard ellipse areas (SEA_B_) in the bidimensional isotopic space of δ^13^C and δ^15^N values of 21 marine invertebrate, fish, and mammal species for two periods (1995–2003 and 2019–2021) of contrasting pressures from anthropogenic and climatic stressors in the St. Lawrence ecosystem. Significant changes confirmed that substantial modifications in community structure have occurred over time, with likely effects on diet composition and energetics of several populations, including endangered species.

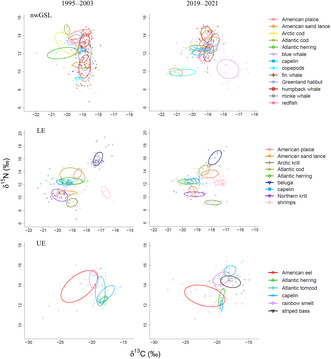

## 1 INTRODUCTION

Anthropogenic activities and climate change have affected marine ecosystems across the globe through significant changes in the physical, chemical, and biological environments (Halpern et al., 2015, 2019). Main stressors include the overexploitation of fish stocks (Myers et al., 1996; Pauly et al., 1998) and bycatch (Komoroske & Lewison, 2015; Lewison et al., 2004), acidification (Hoegh‐Guldberg et al., 2007), sea water warming and oxygen decrease (Breitburg et al., 2018; Gilbert et al., 2005), chemical and acoustic pollution (Cabral et al., 2019; Islam & Tanaka, 2004; Kuşku et al., 2018; Weilgart, 2007), as well as the rapid increase or decrease of top predators (Hammill et al., 2015, 2017, 2021; Lesage, 2021). By affecting habitat quality or species abundance and distribution, these stressors are likely to result in major changes in the trophodynamics of ecosystems, including the degree of competition within and among species, prey–predator relationships, and the diet of species (Bundy et al., 2009; Myers & Worm, 2005; Navia et al., 2012). Understanding how trophic relationships among species and their foraging ecology fluctuate with environmental conditions can help assess the vulnerability and resilience of species to environmental stressors. To assess these changes in trophic and foraging ecology, it is essential to determine the trophic niche baselines.

The realized trophic niche of species (i.e., the niche under limiting factors in their habitat) and the diet can be studied with stable isotope biomarkers (Bearhop et al., 2004; Crawford et al., 2008; Layman et al., 2007). Stable isotopes are also useful to study niche partitioning among and within species (Gavrilchuk et al., 2014; Hobson et al., 2000; Rioux et al., 2022) and, more widely, ecosystem trophic structure (Borrell et al., 2021; MacKenzie et al., 2022). Comparing isotopic niche sizes between species or over time, their position in isotopic space, and overlap with other species, can inform on trophic relationships such as competition or predator–prey relationships, or on changes in space or resource use over time.

Accurate inferences about predator diets require an understanding of how diet and isotopic values of potential prey changed over time and space. Isotopic differences that are observed in a predator may have multiple origins, including a change at the base of the food web (e.g., primary productivity, Suess effect; Bacastow et al., 1996; Keeling, 1979), in the diet of some prey, in the diet of predators or in the relative importance of a prey in the predators' diet (Kurle & McWhorter, 2017; Rodríguez‐Malagón et al., 2021). Documenting long‐term changes in the isotopic signatures of food web components and in the trophic structure of food webs is thus essential to interpret a predator's diet and to forecast how species may cope with environmental stressors.

The Estuary and Gulf of St. Lawrence (hereafter referred to as the EGSL) experienced a major and rapid ecosystem change over the past decades as a result of overfishing, acoustic and chemical pollution, and climate change (Long et al., 2016; Savenkoff et al., 2007; Worm & Myers, 2003). Overfishing in the early 1990s has led to the collapse of Atlantic cod (*Gadus morhua*) and several other commercial demersal fish species (e.g., American plaice *Hippoglossoides platessoides*, Atlantic halibut *Hippoglossus hippoglossus*, and redfish *Sebastes* spp.; Pedersen et al., 2017; Worm & Myers, 2003), but also spring spawning Atlantic herring (*Clupea harengus*; Brosset et al., 2019; LeBlanc et al., 2012), Northern shortfin squid (*Illex illecebrosus*; O'Dor & Dawe, 2013), and American eel (*Anguilla rostrata*; Cairns et al., 2014). As a result, the predation pressure from groundfish species was released on lower trophic levels, leading to an increase in the abundance of species such as Northern shrimp (*Pandalus borealis*), snow crab (*Chionoecetes opilio*), and Greenland halibut (*Reinhardtius hippoglossoides*; Gauthier et al., 2021; Savenkoff et al., 2006). These changes, in turn, led to a change in the community structure, which was previously dominated by large groundfish species, toward a community dominated by invertebrates and small planktivorous (e.g., capelin *Mallotus villosus*, American sand lance *Ammodytes* sp.) and piscivorous (e.g., Atlantic mackerel *Scomber scombrus*) pelagic fishes (Bourdages et al., 2022; Bui et al., 2010; Bundy et al., 2009; Myers et al., 1997; Savenkoff et al., 2007).

In more recent years (2019–2021), the EGSL has been subjected to reduced oxygen concentrations (Gilbert et al., 2005; Jutras et al., 2020), reduced duration and thickness of sea ice cover, and persistent warming of the surface and near‐bottom waters in particular (Galbraith et al., 2022; Plourde et al., 2014; Thibodeau et al., 2010). Most overfished groundfish stocks (e.g., Atlantic cod, American plaice) have remained at low levels (Bourdages et al., 2021; Ricard et al., 2016), and the northern contingent of Atlantic mackerel (Arai et al., 2021; Smith et al., 2021), Northern shrimp (Bourdages et al., 2020a; DFO, 2020a), and Greenland halibut stocks (DFO, 2021) have all declined substantially. In contrast, redfish spp. (Senay et al., 2021), the newly reestablished population of striped bass (*Morone saxatilis*; DFO, 2017), and top predators such as gray seals (*Halichoerus grypus*), harp seals (*Pagophylus groenlandicus*), and harbor seals (*Phoca vitulina*) have all drastically increased in number (Hammill et al., 2021; Mosnier et al., 2023). These changes and those observed in the community composition, local abundance or body condition of zooplankton (Blais et al., 2021; Sorochan et al., 2019) are suspected to have deleterious effects on a number of predators including several endangered species of cetaceans such as blue whales (*Balaenoptera musculus*; Guilpin et al., 2020; Savenkoff et al., 2013), fin whales (*Balaenoptera physalus*; Schleimer et al., 2019), North Atlantic right whales (*Eubalaena glacialis*; Gavrilchuk et al., 2021), and beluga (*Delphinapterus leucas*; Lesage, 2021; Plourde et al., 2014; Williams et al., 2021).

To assess the ecological impacts of climate change and the previously mentioned stressors on vulnerable species, this study examined long‐term changes in the trophic structure of an isotopically well‐documented ecosystem: the EGSL (Cabrol et al., 2021; Gavrilchuk et al., 2014; Hammill et al., 2005; Lesage et al., 2001). More specifically, over 1200 samples collected from 21 species of marine invertebrates, fishes, and mammals during two periods of contrasting anthropogenic (1995–2003) and climatic pressures (2019–2021) were examined for changes in isotopic values, niche breadth and position, and overlap among species. The time periods differed in length and reflect the longer sampling period at the turn of the 21st century compared to the most recent period. These analyses will also provide the baseline required to interpret isotopic values of predators and to assess predator–prey relationships, species diet, and habitat use.

## 2 METHODS

### 2.1 Study area

**FIGURE 1:**
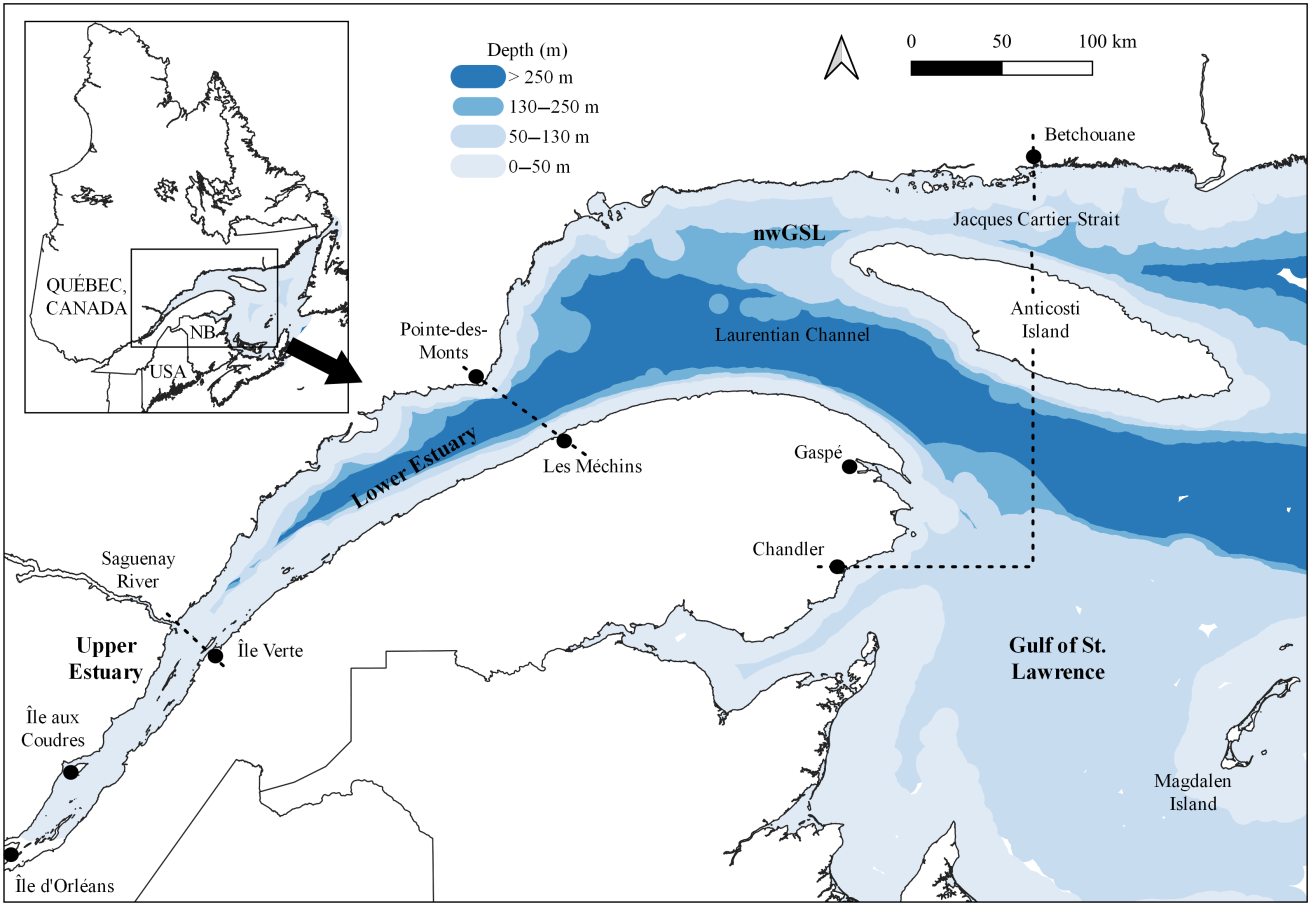
Study area ranging the Upper Estuary (Île d'Orléans to Saguenay River/Île Verte), Lower Estuary (Saguenay River/Île Verte to Pointe‐des‐Monts/Les Méchins), and Gulf of St. Lawrence, Canada. The northwestern gulf (nwGSL) is bounded to the east by the Jacques Cartier Strait near Betchouane and to the south near Chandler. The graduated blue color represents the bathymetry of the Estuary and Gulf of St. Lawrence (from 0 to 520 m deep).

The EGSL in Eastern Canada are among the most productive ecosystems of the world (Figure 1; Dufour & Ouellet, 2007). They connect the Great Lakes Basin, a highly industrialized region, to the Atlantic Ocean. The Upper St. Lawrence Estuary (hereafter referred to as UE) is relatively shallow (generally <30 m deep), varies in salinity from 0 to 25 ppm, and is characterized by strong tidal action, intense water mixing, limited primary productivity, and terrigenous inputs of organic matter (Figure 1; El‐Sabh, 1979; El‐Sabh & Silverberg, 1990; Li et al., 2022). The Lower St. Lawrence Estuary (hereafter referred to as LE) is more heterogeneous in depth, saltier (25–35 ppm), and more productive than the UE as a result of the upwelling of cold and mineral‐rich waters originating from the deep‐water layer of the Gulf of St. Lawrence (hereafter referred to as GSL). The latter is an inland sea connected to the Atlantic Ocean, which is also highly heterogenous in bathymetry, with salinities varying from 25 to 35 ppm (El‐Sabh, 1979; El‐Sabh & Silverberg, 1990). The water column in the LE and GSL is strongly and permanently stratified, allowing the coexistence of temperate and polar species.

### 2.2 Sample collection and preparation

Invertebrate (*n* = 158) and fish (*n* = 733) samples were collected from May to October 1995–2003 (*n* = 598) and 2019–2021 (*n* = 644) in the UE, LE, and northwestern Gulf of St. Lawrence (hereafter referred to as nwGSL, see Figure 1), mainly as part of scientific surveys, but also from commercial fisheries. Briefly, zooplankton species were collected by vertical tows of Bongo nets (1 m diameter × 3 m length) equipped with a 333 μm mesh size, whereas fishes and shrimps were collected using bottom trawls or narrow–weaved wire nets. All samples were frozen in airtight plastic bags at −20°C until preparation and analyses. Zooplankton samples were kept alive overnight before freezing to allow gut clearance, then sorted and identified to the species level in cold filtered sea water. Muscle samples (~1.5 g) were excised from the upper dorsolateral region of each fish specimen; the whole dorsal muscle without the carapace was used for invertebrates, with the exception of calanoid copepods, which were analyzed as whole individuals.

In addition, muscle samples (~500 cm^3^) were collected from 69 relatively fresh beluga carcasses (code <4; Geraci & Lounsbury, 1993) through a long‐term monitoring program (Lesage, 2014). Only samples collected between April and November 1995–2003 and 2019–2021 were used in this study to match the prey data. Samples were excised dorsally near mid‐body length from the side of the carcass sheltered from the sun and frozen in plastic bags at −20°C until processing. While stranded individuals may not represent a healthy population of animals (Cloyed et al., 2023; Payo‐Payo et al., 2013), the use of tissue integrated longer‐term diet likely reduced potential effects of health on isotope ratios. Cloyed et al. (2023) found no difference in stable isotope values between stranded and live dolphin and manatee.

Finally, skin biopsy samples were also obtained from 280 rorquals from four species (i.e., 79 blue whales, 19 minke whales *Balaenoptera acutorostrata*, 85 humpback whales *Megaptera novaeangliae*, and 97 fin whales) between May and November 1995–2003 and 2019–2021, again to match prey sampling. Samples were collected using a crossbow and a hollow‐tipped system while approaching animals from a small vessel (Borobia et al., 1995; Palsbøll et al., 1991). Sampling took place exclusively in the nwGSL, mainly in the Jacques‐Cartier Passage and Gaspé region (Figure 1). The biopsy sampling protocol was reviewed and authorized under the Fisheries Act from 1995 to 2003, and under the Species at Risk Act from 2019 to 2021. The epidermis was separated from the dermis and underlying fat using a sterile scalpel prior to freezing. All rorqual samples from 1995 to 2003 were preserved in a 20% v/v dimethyl sulfoxide (DMSO) solution of deionized water saturated with NaCl with the exception of 5 minke whale samples, which were frozen; all samples from 2019 to 2021 were frozen in plastic vials at −20°C until they were processed. Isotopic values are predictably affected by DMSO preservation and can be reliably restored by standard lipid extraction preceded by multiple water rising, and by applying linear mathematical corrections explicitly developed by Lesage et al. (2010) for rorqual skins (see lipid extraction procedure below and Appendix S1).

All samples (invertebrates, fishes, and mammals) were cut into small pieces, transferred to aluminum cups, freeze‐dried for 48 h at −40°C, and ground to a fine powder using a mortar and pestle (Bosley & Wainright, 1999; Jardine et al., 2003). The freeze‐dried samples were stored in a desiccator until lipid extraction and stable isotope analyses.

### 2.3 Lipid extraction

As lipids are depleted in ^13^C relative to protein and carbohydrate fractions, variability in the lipid content of samples can bias isotopic values (DeNiro & Epstein, 1977; McConnaughey & McRoy, 1979). Stable isotope analyses and the approach to account for lipid effects have improved over the past decades. Since the late 1990s and until the mid‐ to late‐2000s, lipid extraction prior to carbon and nitrogen isotope analyses was the recommended approach (Hobson et al., 1996; Kelly, 2000; Post et al., 2007). However, newer studies have documented an effect of lipid extraction on the δ^15^N values (Mintenbeck et al., 2008; Sotiropoulos et al., 2004; Sweeting et al., 2006), and thus recommended to determine δ^13^C from lipid‐extracted samples and to assess δ^15^N from bulk samples (Kiljunen et al., 2006; Lesage et al., 2010; Logan et al., 2008; Sotiropoulos et al., 2004).

Some samples from 1995 to 2003 (*n* = 553) were analyzed for isotope ratios from the same lipid‐extracted aliquot. To account for lipid effects on δ^15^N values, mathematical corrections were developed for these specific samples in a companion study (Ouellet et al., 2023; see details in Table S1). The effect of lipid extraction on δ^15^N values was toward an enrichment for all 21 species (see Ouellet et al., 2023). In the absence of such correction factors for copepod species, and given the similarity in the carbon to nitrogen elemental ratio (hereafter referred to as CN ratio) between krill and fishes (CN = 3.1 ± 0.1), and copepods (CN = 3.3 ± 0.1), a general equation for krill and fish species was applied to correct copepod values, as recommended by Ouellet et al. (2023, their equation 6). The other samples from 1995 to 2003 (*n* = 45) and those from 2019 to 2021 were analyzed separately: one subsample (bulk) received no further treatment prior to nitrogen isotope analysis, whereas a second subsample (lipid‐free) was lipid‐extracted prior to carbon isotope analysis.

Lipid extraction was conducted using powdered samples (Kelly, 2000; Lesage et al., 2010; Post et al., 2007) and a mixture of chloroform and methanol (2:1 v/v) following the Folch procedure (Folch et al., 1957). Approximately 0.1 g of powdered muscle was mixed with 5 mL of solvent, sonicated for 15 min, and stored overnight at 4°C with gentle shaking on an agitator plate. The mixture was centrifuged at 1500 rpm for 10 min, and lipids from the supernatant were discarded (Folch et al., 1957). This whole procedure was repeated twice, but the mixture was put on the agitator plate only for 1 h instead of overnight between each centrifugation. Samples were dried under a pulsed air evaporator until complete evaporation of liquid (~35 min), oven‐dried overnight at 60°C, and weighed to insure complete drying.

### 2.4 Stable isotope analyses

Subsamples of 0.350–0.600 mg (±0.001 mg) of powdered muscle or skin tissue were weighed in a tin capsule, and analyzed for δ^13^C and δ^15^N values using an Elemental Analyzer EA 1110 CHN coupled to a Delta^Plus^ Isotope Ratio Mass Spectrometer (Environmental Isotope Laboratory and Isotope Tracer Technologies, Waterloo, Ontario). By convention, ^13^C and ^15^N isotope abundances are expressed in delta notation (‰), as δ*X* = [(*R*_sample_/*R*_standard_) − 1] × 1000 where *X* is ^13^C or ^15^N, *R*_sample_ is the corresponding ratio ^13^C/^12^C or ^15^N/^14^N, and *R*_standard_ represents the ratios for their respective standards (i.e., Vienna Peedee Belemnite and atmospheric nitrogen). The accuracy of isotopic analyses, estimated using a commercially certified material (i.e., Acetanilide B2000), was ±0.2‰ for δ^13^C and ±0.3‰ for δ^15^N. Precision, estimated from random duplicate sample analysis (*n* = 401), indicated an average deviation among replicates of 0.2‰ for δ^13^C and δ^15^N.

### 2.5 Suess and laws corrections

To allow the comparison of δ^13^C values of specimens from different periods, δ^13^C values were corrected both for the Laws and Suess effects according to the Subpolar North Atlantic region and for the year 2020 using the library SuessR for R (Clark et al., 2021). The Suess effect is a phenomenon related largely to combustion of fossil fuels and deforestation, and which results in a progressive ^13^C‐depletion of atmospheric carbon dioxide (CO_2_; Bacastow et al., 1996; Keeling, 1979), and a mean ^13^C decrease of dissolved inorganic carbon (hereafter referred as DIC) of the North Atlantic Ocean by approximately −0.026 ± 0.002 per year (Körtzinger et al., 2003). The δ^13^C values were adjusted for the Suess effect, resulting in corrections ranging from −0.7‰ for year 1995 to 0‰ for recent years (2020 and 2021). The Laws effect represents the change in δ^13^C values related to the effect of water temperature on aqueous CO_2_ concentrations and carbon fractionation by phytoplankton over thousands of years (Laws et al., 2002). As a result, corrections for this effect were small, ranging from −0.016‰ (for year 1995) to 0.001‰ (for years 2020 and 2021).

### 2.6 Data analysis

Overall, 21 species were available for both time periods, with the exception of striped bass that were available only for the recent period (2019–2021) due to the fact that this population was reintroduced in 2003 in the UE (DFO, 2017). In the absence of knowledge of movements and habitat use before sampling, each organism was assigned to a region (i.e., nwGSL, LE or UE) based on sampling location, although some organisms may have moved among study regions or may have spent time outside the study area prior to sampling. Rorquals, for instance, were sampled in the nwGSL during summer, but may have visited other parts of the Gulf or the LE prior to sampling (Lesage et al., 2017; Schleimer et al., 2019). Belugas use the St. Lawrence Estuary (hereafter referred to as the SLE) and part of the Saguenay Fjord as their core area during spring, summer, and fall, but are known to move among zones on a daily or at least a seasonal basis, with part of the population using the nwGSL during the fall and winter (Bonnell et al., 2022; Mosnier et al., 2010; Ouellet et al., 2021; Simard et al., 2023; Vladykov, 1944). Belugas sampled through beach‐cast carcasses could not be assigned with certainty to a specific region within the SLE (Truchon et al., 2013), and were all assigned to the LE, although some may have used the UE intensively. Species of fish such as Atlantic cod (Castonguay et al., 1999), American eel (Béguer‐Pon et al., 2014), and Atlantic herring (Couillard et al., 2022) may overwinter outside our study area (e.g., the southern GSL, northeastern GSL, Newfoundland), but migrate into the nwGSL or the SLE to spawn or forage during the spring, summer, and fall. Nevertheless, these species were assumed to have remained within the region where they were caught during most of the study period (i.e., late April–October), and thus to have integrated the isotopic signatures of the regions studied.

### 2.7 Statistical analyses

Ontogeny may affect stable isotope signatures (e.g., Lesage et al., 2001; Peters et al., 2020), with young stages having different diets or being distributed differently in the water column or in different locations (e.g., McAllister et al., 2021; Senay et al., 2021; Walkusz et al., 2020). A previous study (i.e., Lesage et al., 2001) and a preliminary analysis of our samples revealed an effect of total length on δ^13^C and δ^15^N values in some of the species sampled (Tables S2 and S3). To account for this effect and to ensure a similar distribution of size classes among periods, specimens of similar size classes were used, and only adult stages were kept for all species (see details in Appendix S1). The representation of the size distribution was verified with a density plot using the ggplot 2 package (Wickham, 2016).

As expected from a previous study (Lesage et al., 2001), geographical location of collection and species significantly affected the δ^13^C and δ^15^N values in our study area. As a result, generalized linear models (GLMs) with Gaussian distribution were used to test for differences in δ^13^C and δ^15^N values among species, the three regions, two periods, and the 3‐way interaction. Only species present in the three regions and sampled in both periods were selected for this specific analysis (i.e., Atlantic cod, capelin, Atlantic herring, American plaice, and American sand lance, *n* = 480). The normality of residuals was assessed using normal quantile–quantile plots and we assessed heteroscedasticity by plotting residuals versus fitted values and versus each covariate (species, period, and region; Quinn & Keough, 2002; Zuur et al., 2010).

Potential changes in isotopic signatures of species between periods (2019–2021 vs. 1995–2003) were examined for each element and region separately using GLMs with Gaussian distribution using species and period as covariates. Pairwise comparisons were conducted using the library emmeans (Lenth, 2022) while applying a Bonferroni correction to the alpha level to account for the number of comparisons. Model assumptions were verified by plotting residuals versus fitted values and each covariate, and by using a normal quantile–quantile plots (Quinn & Keough, 2002; Zuur et al., 2010).

As isotopic signatures varied over two axes simultaneously, a stable isotope trajectory analysis (hereafter referred to as SITA; Sturbois et al., 2021) was used to quantify changes between periods. SITA is a powerful tool to explore and quantify isotopic shifts, as it allows the direction of shifts in the bidimensional isotopic space (δ‐space) to be represented and tested for significance. The net change in isotopic signatures between periods for a given region and species was expressed through trajectory roses (Sturbois et al., 2021), and was calculated as the Euclidean distance between the signatures in 1995–2003 and 2019–2021, and the angle α of the shift between the two periods (0–360° direction in the δ‐space; Sturbois et al., 2021).

Niche breadth for the different species was examined using two metrics: the standard ellipse area corrected for small sample size (SEA_C_) and the Bayesian standard ellipse area (SEA_B_) and its 95% credible interval (95% CI) occupied in the bidimensional isotopic space. Both were estimated using the library SIBER (Jackson, 2019). These two metrics take sample size into account when estimating ellipse breadth, although the latter tends to stabilize around a sample size (*n*) of 30; if *n* is small, then SEA_C_ may be more uncertain (i.e., wider credibility intervals). The SEA_C_ and SEA_B_ were set to contain 40% of the data and both represent the core isotopic niche, that is, resources most often used (Jackson et al., 2011). The posterior distributions of SEA_B_ were used to estimate the probability of a change in niche breadth between periods, that is, the probability of the posterior distribution of SEA_B_ being smaller in the recent period (2019–2021) compared to the past period (1995–2003). However, the SEA_C_ was used to assess overlap in the overall isotopic niche between periods and for each species and region separately, as it allows for niche breadth overlap and position in the δ‐space to be assessed (Jackson et al., 2011). The overlap between species ellipses for a particular period and region were calculated as the proportion of the total SEA_C_ of species A and species B that overlap with each other, where a value of 0% indicates no overlap and a value of 100% indicates complete niche overlap. This latter index was used to document changes in community structure between periods and identify potential competitors. All statistical analyses were performed using R software version 4.2.1 (R Development Core Team, 2017).

## 3 RESULTS

Isotopic values varied among invertebrate, fish, and mammal species from −27.8‰ to −13.0‰ for δ^13^C, and from 6.7‰ to 20.4‰ for δ^15^N (Figure S1, Table S4). Given the absence in our sample of some top predators such as seals, and of the smaller zooplankton species and phytoplankton, this range does not represent the full extent of the regional food web. Generalized linear models examining variation in δ^13^C and δ^15^N values were highly significant (both *p* < .001), and the total variance explained by fixed effects (i.e., species, region, and period) was relatively high for δ^13^C (*R* ^2^ = .57) and for δ^15^N (*R* ^2^ = .80). The 3‐way interaction was highly significant for both δ^13^C and δ^15^N (GLMs, *F*_5,455_ = 19.8 and 16.1, respectively; both *p* < .001), confirming the need for taking into account regions, species, and periods in subsequent analyses (see details in Appendix S1).

### 3.1 Isotopic signature changes

**FIGURE 2:**
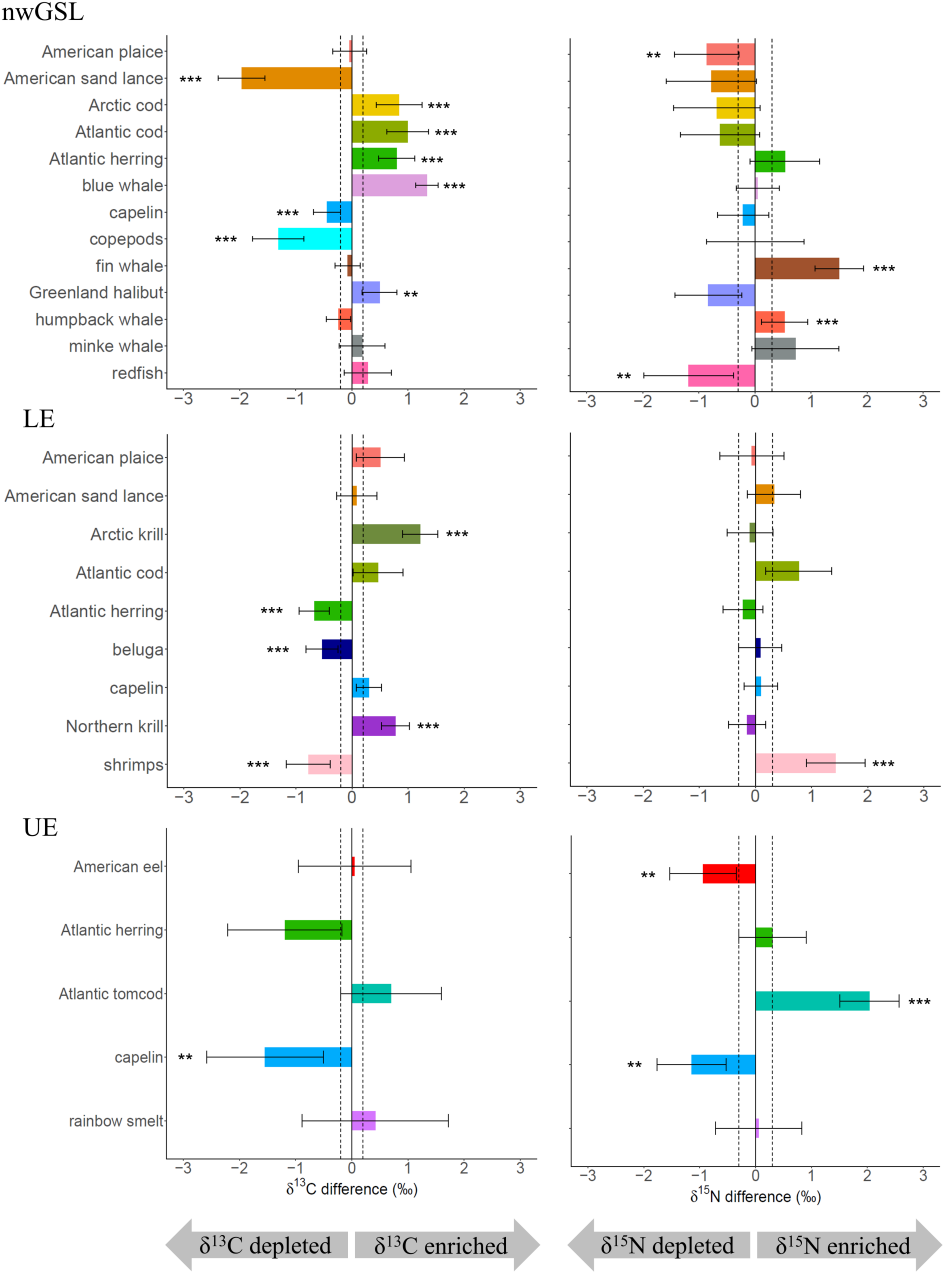
Amplitude and direction of changes observed in δ^13^C and δ^15^N values during the 2019–2021 period relative to 1995–2003 for various species in the northwestern Gulf of St. Lawrence (nwGSL), and the Lower (LE) and Upper (UE) St. Lawrence Estuary regions. Significance of change (pairwise comparisons with a Bonferroni correction, *p* < .004 for nwGSL, *p* < .006 for LE, *p* < .01 for UE) is indicated by asterisks (**p < .01, ***p < .001). The notations “δ^13^C and δ^15^N depleted” mean a depletion in the recent period compared to the past period, while “δ^13^C and δ^15^N enriched” means the opposite. The dotted line represents the isotopic analytical errors.

Overall, all but two (i.e., minke whale, rainbow smelt *Osmerus mordax*) of the 20 species exhibited a significant change in isotopic signatures (either a single element or both) between 1995–2003 and 2019–2021 (Figure 2, Tables S4 and S5). However, the direction of change (i.e., depletion or enrichment) was inconsistent among species and sometimes also among regions for the same species (Figure 2, Tables S4 and S5). In the nwGSL for instance, a decrease in δ^13^C values was observed at the base of the food web (i.e., copepods) and in American sand lance and capelin, whereas an increase in δ^13^C was noted for Arctic and Atlantic cod, Atlantic herring, Greenland halibut, and blue whales (Figure 2, Table S5). In the LE, the tendency of change in isotopic signatures at the base of the food web was variable among species (e.g., an enrichment in ^13^C for Arctic and Northern krill, and a ^13^C‐depletion and ^15^N‐enrichment for shrimps), whereas at higher trophic levels, changes observed were generally toward a decreased in δ^13^C values (observed for Atlantic herring and beluga; Figure 2, Tables S4 and S5). In the UE, some species such as capelin underwent a major reduction in both δ^13^C and δ^15^N values, whereas other species showed changes in a single isotope (e.g., enrichment in ^15^N for Atlantic tomcod and depletion in ^13^C for Atlantic herring; Figure 2, Tables S4 and S5). In general, a larger number of species showed a difference in δ^13^C values compared to δ^15^N between periods (Figure 2, Table S5). Effect size, however, was not necessarily less for δ^15^N than for δ^13^C values (change between −1.2‰ and 2.0‰ for nitrogen, and between −2.0‰ and 1.3‰ for carbon).

**FIGURE 3:**
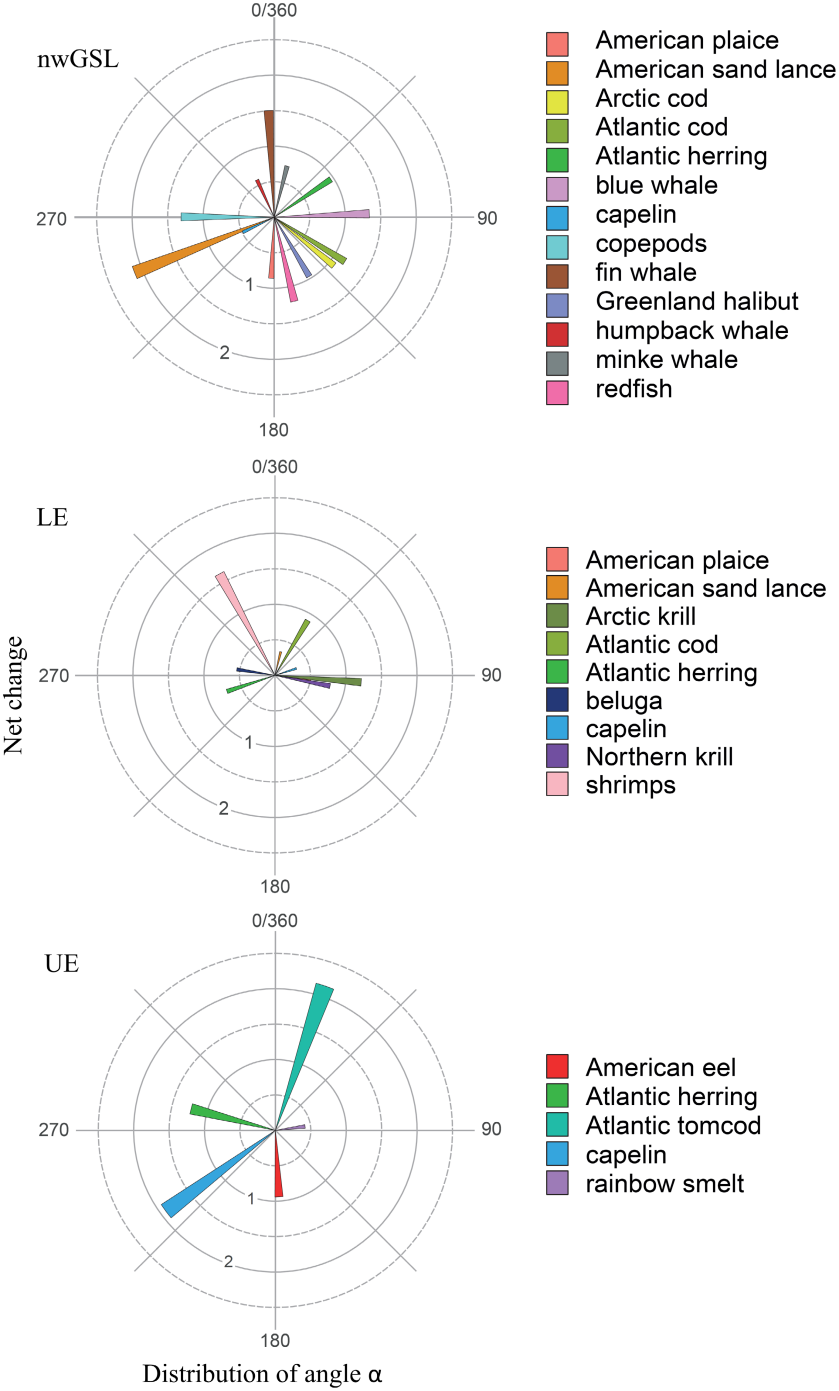
Trajectory roses for the northwestern Gulf of St. Lawrence (nwGSL), and the Lower (LE) and Upper (UE) St. Lawrence Estuary regions. Angles *α* and net changes are represented in the trophic trajectory rose for each species and represent the difference between isotopic values of the 2019–2021 period compared to the 1995–2003 period. Net changes (0–2.5) represent the magnitude of change, and it is determined with Euclidean distance. Angle *α* values (0–360°) represent the nature of change in the bidimensional isotopic space (0–90°: +δ^13^C and +δ^15^N; 90–180°: +δ^13^C and −δ^15^N; 180–270°: −δ^13^C and −δ^15^N; 270–360°: −δ^13^C and +δ^15^N).

When combining the two isotopes to better express isotopic shift between periods, a net change higher than 1.0‰ was observed for nine of the thirteen species in the nwGSL, two of the nine species in the LE, and three of the five species in the UE (Figure 3, Table S4). Again, the amplitude and angle direction of change in the δ‐space was not necessarily consistent among species nor among regions for a particular species (Figure 3, Table S4).

### 3.2 Niche breadth and position

**FIGURE 4:**
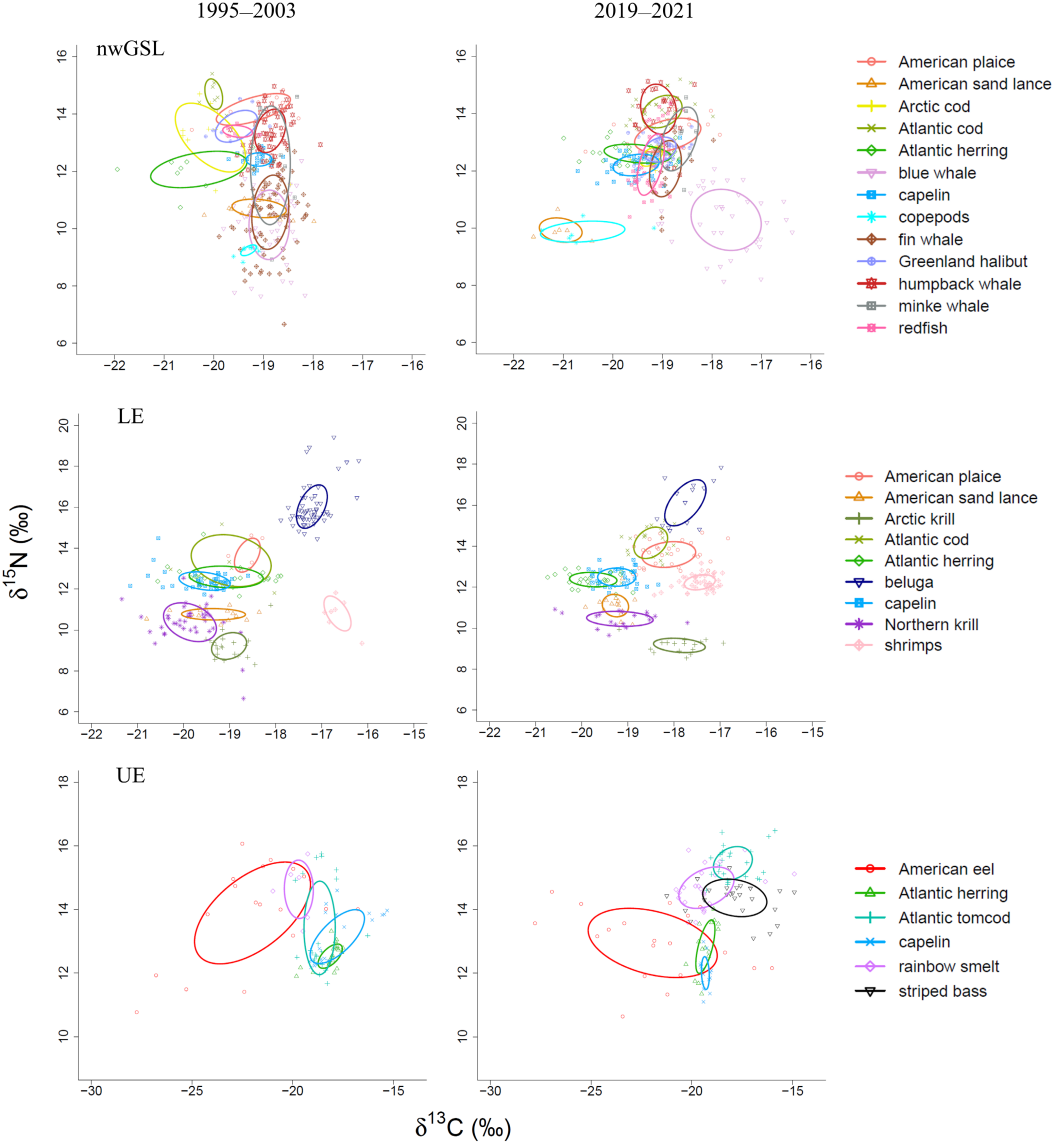
Bayesian standard ellipse areas (SEA_B_) in the bidimensional isotopic space of δ^13^C and δ^15^N values of various species for both periods in the northwestern Gulf of St. Lawrence (nwGSL), and the Lower (LE) and Upper (UE) St. Lawrence Estuary regions. SEA_B_ contains 40% of the data. δ^13^C and δ^15^N values are plotted for each individual according to period (ο 1995–2003, Δ 2019–2021).

When translated into niche metrics, these isotopic changes resulted in a niche shift in the δ‐space, such that niches overlapped little (<30%) or not at all (0%) between periods in most species (Table 1). Species with the smallest shifts in niche (14.9%–29.4%) between periods were the humpback and minke whales in the nwGSL, American sand lance and capelin in the LE, and American eel and rainbow smelt in the UE. The SEA_B_ indicated that this shift was also accompanied by a change in niche breadth for some of the species. While niche breadth remained unchanged over time for 16 of the 27 region‐specific species, a significant niche size reduction in the recent period (2019–2021) compared to the past period (1995–2003) was noted for three of the 13 species of the nwGSL (i.e., Arctic cod, Atlantic herring, and fin whales), two of the nine species of the LE (i.e., Atlantic cod and herring), and two of the five species of the UE (i.e., Atlantic tomcod and capelin; Table 1, Figure 4, Figures S2 and S3). Change toward a niche size expansion in the recent period was observed only in the nwGSL and for four of the 13 species (i.e., Atlantic cod, capelin, copepods, and redfish; Table 1, Figure 4, Figures S2 and S3).

**TABLE 1:** Sample size (*n*), standard ellipse area corrected for small sample size (SEA_C_), and Bayesian standard ellipse area (SEA_B_) and its 95% credible interval (95% CI) calculated from various species for both periods for the northwestern Gulf of St. Lawrence (nwGSL), and the Lower (LE) and Upper (UE) St. Lawrence Estuary regions.

Species	1995–2003			2019–2021			Probability	Relative overlap (%)
	*n*	SEA_C_ (‰^2^)	SEA_B_ [95% CI] (‰^2^)	*n*	SEA_C_ (‰^2^)	SEA_B_ [95% CI] (‰^2^)		
nwGSL								
American plaice *Hippoglossoides platessoides* (13.4–41.0)	10	1.2	1.1 [0.6: 1.8]	20	1.1	1.1 [0.7: 1.7]	.50	0.8
American sand lance *Ammodytes* sp. (8.3–15.5)	17	0.5	0.5 [0.3: 0.8]	6	0.6	0.4 [0.2: 1.1]	.56	0.0
Arctic cod *Boreogadus saida* (10.5–17.6)	9	2.2	1.8 [0.9: 4.0]	10	0.2	0.2 [0.1: 0.4]	**1.00**	0.0
Atlantic cod *Gadus morhua* (25.1–46.6)	7	0.3	0.2 [0.1: 0.5]	30	0.7	0.7 [0.5: 0.9]	**.02**	0.0
Atlantic herring *Clupea harengus* (16.3–30.3)	10	1.8	1.5 [0.8: 3.0]	29	0.7	0.6 [0.4: 0.9]	**1.00**	0.6
Blue whale *Balaenoptera musculus*	44	1.6	1.5 [1.1: 2.0]	35	2.1	2.0 [1.4: 2.9]	.10	0.0
Capelin *Mallotus villosus* (10.2–16.3)	21	0.2	0.2 [0.1: 0.3]	38	0.5	0.5 [0.4: 0.7]	**.00**	4.0
Copepods *Calanus finmarchicus*, *C. hyperboreus*, *Metridia longa*, *Paraeuchaeta norvegica*	10	0.1	0.1 [0.0: 0.1]	6	0.7	0.5 [0.2: 1.5]	**.00**	0.0
Fin whale *Balaenoptera physalus*	78	1.5	1.5 [1.2: 1.8]	19	1.0	0.9 [0.6: 1.4]	**.96**	3.8
Greenland halibut *Reinhardtius hippoglossoides* (21.9–37.6)	10	0.7	0.6 [0.3: 1.1]	40	0.4	0.4 [0.3: 0.6]	.87	0.0
Humpback whale *Megaptera novaeangliae*	62	0.7	0.7 [0.5: 0.9]	23	0.9	0.9 [0.5: 1.4]	.18	14.9
Minke whale *Balaenoptera acutorostrata*	10	2.6	2.2 [1.0: 4.3]	9	1.2	1.0 [0.4: 2.1]	.77	19.3
Redfish *Sebastes* spp. (21.5–30.5)	5	0.2	0.1 [0.1: 0.4]	40	0.8	0.8 [0.6: 1.1]	**.00**	0.0
LE								
American plaice *Hippoglossoides platessoides* (18.4–39.6)	6	0.7	0.5 [0.2: 1.3]	30	1.1	1.0 [0.7: 1.6]	.09	7.0
American sand lance *Ammodytes* sp. (9.6–15.4)	14	0.6	0.5 [0.3: 0.9]	15	0.5	0.4 [0.3: 0.8]	.70	15.0
Arctic krill *Thysanoessa* spp.	20	0.8	0.8 [0.5: 1.2]	16	0.7	0.7 [0.4: 1.1]	.83	0.0
Atlantic cod *Gadus morhua* (23.0–47.5)	6	3.4	2.4 [1.0: 6.3]	22	0.8	0.8 [0.5: 1.2]	**1.00**	5.7
Atlantic herring *Clupea harengus* (18.5–32.9)	26	1.3	1.2 [0.8: 1.8]	26	0.6	0.5 [0.4: 0.8]	**1.00**	6.3
Beluga *Delphinapterus leucas*	55	1.0	1.0 [0.7: 1.3]	14	1.3	1.2 [0.7: 2.1]	.19	1.0
Capelin *Mallotus villosus* (11.5–17.3)	43	0.8	0.7 [0.5: 1.0]	33	0.6	0.5 [0.4: 0.8]	.87	22.2
Northern krill *Meganyctiphanes norvegica*	40	1.0	1.0 [0.8: 1.2]	18	0.9	0.8 [0.5: 1.3]	.83	5.7
Shrimps *Pandalus borealis*, *P. montagui* (9.3–16.9)	7	0.7	0.5 [0.2: 1.2]	41	0.4	0.4 [0.3: 0.5]	.90	0.0
UE								
American eel *Anguilla rostrata* (58.0–104.0)	18	12.0	11.0 [6.8: 18.2]	19	10.2	9.2 [5.8: 15.3]	.72	22.4
Atlantic herring *Clupea harengus* (14.5–25.4)	22	0.6	0.6 [0.4: 0.9]	15	1.0	0.9 [0.5:1.5]	.06	0.0
Atlantic tomcod *Microgadus tomcod* (14.5–33.0)	21	3.6	3.3 [2.1: 5.2]	26	1.5	1.5 [1.0: 2.1]	**1.00**	0.0
Capelin *Mallotus villosus* (11.7–15.5)	20	2.7	2.4 [1.6: 4.0]	15	0.3	0.3 [0.2: 0.5]	**1.00**	0.0
Rainbow smelt *Osmerus mordax* (14.7–22.5)	7	2.1	1.5 [0.7: 3.7]	25	2.6	2.5 [1.6: 3.6]	.22	29.4
Striped bass *Morone saxatilis* (18.7–25.3)	–	–	–	24	2.9	2.7 [2.3: 3.1]	–	–

*Note*: Latin names and body length (in cm) are indicated after species common names. The probability is that of the posterior distribution of SEA_B_ being smaller in the recent period (2019–2021) compared with the past period (1995–2003) and significance of change (probability <.05 or >.95) is indicated in bold. The relative overlap (%) represents the isotopic niche overlap between periods for a given species. A reduction in niche breadth in the recent period compared with the past period is indicated in blue while an increase in niche breadth is indicated in orange.

### 3.3 Community structure

In the nwGSL, major changes in niche overlap among species were noted over time. During the most recent period for instance, American sand lance and copepods showed a greater niche differentiation from other species compared to the earlier period (Figure 4, Table 2a). Similarly, while the niche of blue whales overlapped with that of fin and minke whales in the 1995–2003 period, this overlap vanished in the most recent period. Overall, ten of the 13 species showed overlap with a higher number of species during the most recent period compared to 1995–2003 (Figure 4, Figure S2, Table 2a). Atlantic cod, which did not overlap with any sampled species in 1995–2003, shared their larger niche with humpback whales during the most recent period. Some pelagic (i.e., Arctic cod, Atlantic herring, and capelin) and demersal fish species (i.e., American plaice, Greenland halibut, and redfish), and two of the whale species (i.e., fin and minke whales) overlapped more extensively among each other in the recent period compared to the previous one (Table 2a). Atlantic herring, Greenland halibut, and redfish were the species that shared their niches with the largest number of species (5 or 6 species); copepods, American sand lance, Atlantic cod, and blue and humpback whales were taxa that overlapped the least with other species (0–1 species).

**TABLE 2:** The core isotopic niche overlap between species represents the proportion of niche of species A and species B that overlap with each other, where a value of 0% indicates no overlap and a value of 100% indicates complete overlap of niches, for the northwestern Gulf of St. Lawrence (nwGSL), and the Lower (LE) and Upper (UE) Estuary in both periods.

Species	American plaice	American sand lance	Arctic cod	Atlantic cod	Atlantic herring	Blue whale	Capelin	Copepods	Fin whale	Greenland halibut	Humpback whale	Minke whale	Redfish
a. nwGSL													
American plaice		0	0	0	0	0	0	0	0	10.2	0.1	0.3	0
American sand lance	0		0	0	0	12.6	0	0	16.5	0	0	3.9	0
Arctic cod	0.1	0		0	6.0	0	0	0	0	4.5	0	0	0.4
Atlantic cod	0.2	0	0		0	0	0	0	0	0	0	0	0
Atlantic herring	0	0	18.4	0		0	0	0	0	0	0	0	0
Blue whale	0	0	0	0	0		0	0	33.9	0	0	6.7	0
Capelin	0	0	8.4	0	9.1	0		0	0	0	0	5.9	0
Copepods	0	20.6	0	0	0	0	0		0	0	0	0	0
Fin whale	1.8	0	0.5	0	2.5	0	0.9	0		0	0	13.1	0
Greenland halibut	11.5	0	16.6	0	12.7	0	0	0	1.0		0	0	18.3
Humpback whale	2.9	0	0	36.2	0	0	0	0	0	0		15.5	0
Minke whale	23.8	0	0	0	0.3	0	0	0	10.0	7.7	0		0
Redfish	4.6	0	20.0	0	12.0	0	12.8	0	8.9	17.0	0	0	

Species	American plaice	American sand lance	Arctic krill	Atlantic cod	Atlantic herring	Beluga	Capelin	Northern krill	Shrimps
b. LE									
American plaice		0	0	12.2	0	0	0	0	0
American sand lance	0		0	0	0	0	0	5.2	0
Arctic krill	0	0		0	0	0	0	0	0
Atlantic cod	3.7	0	0		14.4	0	0	0	0
Atlantic herring	0	0	0	0		0	16.5	0	0
Beluga	0	0	0	0	0		0	0	0
Capelin	0	0	0	0	8.5	0		0	0
Northern krill	0	3.4	0	0	0	0	0		0
Shrimps	0	0	0	0	0	0	0	0	

Species	American eel	Atlantic herring	Atlantic tomcod	Capelin	Rainbow smelt	Striped bass
c. UE						
American eel		0	0	0	5.8	–
Atlantic herring	3.2		2.2	9.5	0	–
Atlantic tomcod	0	0		12.3	0	–
Capelin	0	2.6	0		0	–
Rainbow smelt	0	0	0	0		–
Striped bass	11.6	0.1	22.5	0	33.7	

*Note*: The 1995–2003 period is represented at the top of the diagonal, and the 2019–2021 period is represented at the bottom of the diagonal. A reduction in overlap in the recent period compared to the past period is indicated in blue while an increase in overlap is indicated in orange.

In other regions (i.e., LE and UE) where a lesser number of species were sampled, when a change was noted during the most recent period, there was a tendency toward a reduction in niche breadth and overlap (Table 2b,c). More specifically, in the LE, only Atlantic cod and herring showed a reduction in niche breadth and overlap over time; beluga, Arctic krill, and shrimps showed unchanged niche breadth over time and overlapped with no other species regardless of periods. In the UE, the most affected species were the marine pelagic fishes (i.e., Atlantic herring, Atlantic tomcod, and capelin) for which a decrease in niche size and overlap between them was noted (Figure 4, Figure S2, Table 2c). However, the striped bass has a significant overlap with Atlantic tomcod (22.5%) and rainbow smelt (33.7%) over the recent period.

## 4 DISCUSSION

Our sample of over 1200 marine invertebrates, fishes, and mammals spanning two periods of contrasting anthropogenic and climatic pressures indicated long‐term changes in the trophic structure of the St. Lawrence marine ecosystem. Significant temporal changes were noted in the isotopic signature and niche position of most species, which were accompanied in several species by a change in niche breadth and degree of niche partitioning among species. The direction of these changes and effect sizes were inconsistent among species, suggesting no systematic modification of the ecosystem over time. The observed patterns may have been caused by a change at the base of the food web, in the diet composition of a predator or the diet of some of their prey. Each of these potential causes needs to be considered and carefully evaluated to accurately interpret temporal variations in a predator's isotopic signature, isotopic niche or diet.

### 4.1 Changes in stable isotope signatures

The base of the food web can be affected isotopically by the Laws and Suess effects, which we accounted for in our study (Durante et al., 2021; Graham et al., 2010; Radabaugh et al., 2013). Phytoplankton isotopic signature is affected by phytoplankton types, size, growth rate, water temperature and concentrations of DIC, and dissolved inorganic nitrogen (hereafter referred to as DIN). Phytoplankton use DIC to fuel photosynthesis. At high productivity, discrimination against the heavier isotope decreases, leading to an increase in phytoplankton δ^13^C values (Laws et al., 1995; Rau et al., 1996; see also supplementary material in Gavrilchuk et al., 2014). Similarly, primary producers are influenced by the isotopic signature of DIN, which can take several forms in the marine environment (i.e., urea, nitrate, and ammonium), and by associated metabolic pathways (i.e., denitrification or atmospheric nitrogen fixation processes; Cline & Kaplan, 1975; Graham et al., 2010; Trueman et al., 2019). As is the case for carbon isotopes, seasonal and annual changes in nutrient availability and photosynthesis rates affect δ^15^N value of primary producers (Kurle & McWhorter, 2017) and propagate to higher trophic level.

Primary consumers have often been used as the isotopic baseline for ecosystems, as they integrate some of the isotopic variability from primary producers (Post, 2002; Vander Zanden & Rasmussen, 2001) and are not as prone as phytoplankton to contamination by other particles (Schell et al., 1998). Our study did not include POM or phytoplankton samples, but relied on copepods (*Calanus* spp.) and the Arctic krill (*Thysanoessa* spp.). While both taxa feed on phytoplankton, they also consume zooplankton to a variable extent (Cabrol et al., 2018; Kozak et al., 2020; Mayor et al., 2006; Ohman & Runge, 1994). Arctic krill (and Northern krill) showed an increase in δ^13^C values during the most recent period, with blue whales, which feeds exclusively on euphausiids (Gavrilchuk et al., 2014), echoing this trend. In contrast, copepods showed a notable reduction in δ^13^C values during the most recent period, with American sand lance, mainly a consumer of copepods (Staudinger et al., 2020), and capelin (Chamberland et al., 2022; Dalpadado & Mowbray, 2013) both echoing this trend. However, other secondary consumers (e.g., Arctic cod, Atlantic herring, and fin whales) known to feed to some extent on copepods and euphausiids but also on other species (Cabrol et al., 2021; Darbyson et al., 2003; Hanson, 2018; Nelson et al., 2020), varied in the direction of their isotopic shifts during the most recent period, challenging the possibility of a shift in the ecosystem baseline. A study tracking parallel changes in DIC and DIN concentrations and in isotopic signature of community constituents up to secondary consumers over time indicated that isotopic variations attenuate rapidly with trophic level (Gavrilchuk et al., 2014). Gavrilchuk et al. (2014) suggest that short‐ to medium‐term (i.e., months) fluctuations of the food web baseline become of diminishing concern for interpreting diet and trophic position as species move away from the baseline and increase in trophic position. While a link between an isotopic shift in the ecosystem baseline and primary consumers cannot be ruled out, it seems more likely based on the Gavrilchuk et al. (2014) study, that the isotopic variations observed in many secondary and tertiary consumers is due mainly to a change in diet or habitat use, and not only to a simple baseline shift. However, this hypothesis needs to be further tested, for example through amino acid compound‐specific isotopic analysis (CSIA).

Whether a shift in a consumer's isotopic signature is partly or entirely due to a change in its own diet composition or to a change in the diet of its prey cannot be determined without an exhaustive analysis of the diet composition of each community component using quantitative models. Generally, a change in diet composition (either for a predator or their prey) should be detected statistically more easily via δ^15^N values than δ^13^C values given the larger trophic enrichment associated with nitrogen isotopes (~3‰ vs. ~1‰ for carbon; Caut et al., 2009; DeNiro & Epstein, 1978, 1981; Minagawa & Wada, 1984; Peterson & Fry, 1987). Given that both elements show an enrichment with trophic level, a parallel change in the two elements may indicate ingestion of new prey items from a different trophic position within the same ecosystem. The absence of a concomitant change of the two isotopes is trickier to interpret: a change in δ^13^C but constancy in δ^15^N might express a shift in the predator habitat use without a change in diet or the consumption of new prey items occupying the same trophic level as the previous prey but issued from a different carbon sources (i.e., pelagic vs benthic). Alternatively, constancy in δ^13^C with a change in δ^15^N might reflect a switch to prey from different trophic positions without a change in habitat use. Twelve species showed a significant change in δ^13^C values between periods that was unaccompanied by a change in δ^15^N values, whereas eight others showed the reversed pattern.

Major changes have been documented over the past decades in the physical oceanography of the EGSL ecosystem (Galbraith et al., 2022; Jutras et al., 2020; Plourde et al., 2014; Thibodeau et al., 2010), with consequences on species community assemblage and abundance (Blais et al., 2021; Bourdages et al., 2022; Bui et al., 2010; Plourde et al., 2014). For instance, a substantial decline in abundance was noted for Greenland halibut (Gauthier et al., 2021; Ghinter et al., 2021), Northern shrimp (Bourdages et al., 2020b), and Atlantic cod (Brassard et al., 2020), and more recently the spring spawning Atlantic herring stocks (Brosset et al., 2019; MPO, 2022). Meanwhile, several other species have seen major increases in their abundance, including a newly reestablished population of striped bass where it has been reintroduced in 2003 in the UE (DFO, 2017), redfish spp. (Bourdages et al., 2022; DFO, 2020b), and several seal species (Hammill et al., 2017, 2021; Mosnier et al., 2023). These changes in species abundance have likely resulted in effects on diet composition or habitat use for some of the species and their potential prey. While a full exploration of the diet of each of the sampled species was beyond the scope of the current study, an interpretation of patterns in niche breadth change and degree of overlap was attempted.

### 4.2 Community structure

Changes in niche breadth were observed for several species between periods, with some species showing a decrease and others an increase (all observed in the nwGSL) in niche breadth. These observations suggested that some species increasingly focused on a reduced set of prey or habitat types during the most recent period, either individually or as a population (Ercoli et al., 2014; Layman et al., 2007), while others instead diversified their diet or habitat types, again, at the individual or population level. The observed adaptation of a species's niche size and its persistence in the EGSL ecosystem suggest some resilience to ecosystemic changes (Schmidt et al., 2011). When resources are unlimited (i.e., there is no competition, only resource sharing), pressure on niche breadth is not as constrained by other species, allowing more flexibility in niche size and resource use (Booth & Murray, 2019). Under these conditions, there can be some overlap between niches without compromising the coexistence of species (MacArthur & Levins, 1967; Polechová & Storch, 2018). Under the niche theory, competition works when resources are scarce or limited, niche overlap between ecologically similar species must be the lowest so as to allow species to coexist by reducing competition from concurrent exploitation of the same resources (Pianka, 1974; Schoener, 1982). A reduction in competition can be achieved through a diversification of the diet or habitat used, or by exploiting only a portion of the niche available under the no‐competition conditions (Baltensperger et al., 2015).

This study indicates a stronger ecological niche partitioning in recent years, as revealed by the relatively small (<30%) overlap of SEA_B_ among species (Ceia et al., 2023; Golikov et al., 2020; Langton, 1982). All species where a decrease in niche size was observed over time adhered to this pattern, where the decrease in niche breadth was concomitant to a decrease in overlap with potential competitors (Tables 1 and 2). An interesting example was the fin whale, mainly a krill eater in the past (Cabrol et al., 2021; Gavrilchuk et al., 2014), and which increased trophic position significantly (+1.5‰) in the recent period, reducing overlap with other sympatric rorqual species (blue and minke whales) while maintaining a niche distinct from the humpback whales. Whether or not part of this signal results from the earlier arrival noted for this species (and humpback whales) in the GSL is unknown (Ramp et al., 2015). Another notable example concerns Atlantic tomcod, Atlantic herring, and capelin in the UE: these species from this relatively small habitat of brackish waters all showed a decrease in niche size and overlap during the most recent period, suggesting a possible shrinkage of resources in this area. Whether this observed niche contraction is due to the reintroduction of a known competitor (i.e., striped bass), is unknown. The increasing abundance of this apex predators, especially in the UE, and of different species of seals in the EGSL in recent years (DFO, 2017; Hammill et al., 2015, 2017, 2021; Mosnier et al., 2023), could have increased competition for prey or changed habitat use due to predation risk. A reduction in niche breadth of consumer prey as a result of top‐down control has been observed in other species such as lizards (Pringle et al., 2019). There were, however, two notable exceptions to the above pattern: Arctic cod and Atlantic herring from the nwGSL. These two species overlapped slightly (6%) in their niches during 1995–2003. They both underwent a niche size reduction over time, but that was accompanied by an increase and not a decrease in niche overlap (18%). These observations suggest that these two species may be exploiting a similar and reduced set of prey in recent years, increasing the potential for competition. In addition, the niche of these species also increased in overlap with that of capelin, redfish, American plaice, Greenland halibut, fin and minke whales, and thus their potential competitors.

In contrast, a niche expansion during the most recent period was noted for four species, all from the nwGSL: Atlantic cod, redfish, capelin, and copepods. While Atlantic cod and copepods did not overlap in their niche with any of the sampled species in either period, redfish and capelin shared their niches with a larger number of species in recent years, although without increasing overlap with the species they shared their niches with during 1995–2003. Moreover, the unprecedented increase in abundance of redfish spp. in recent years may explain the large increase in its niche breadth and overlap with other species over time (Bourdages et al., 2022; DFO, 2020b).

Overall, the two endangered/threatened marine mammals (i.e., beluga and blue whales) showed little overlap with other species. For instance, the endangered blue whales has shown a separation of its niche from those of American sand lance, fin and minke whales during the recent period. Blue whale sightings decreased since the 1980s in some areas of the nwGSL (Comtois et al., 2010; Ramp & Sears, 2013). A decrease in sightings may be associated with a reduced abundance or availability of preferred prey in the GSL (i.e., Arctic krill; McQuinn et al., 2015; see also Guilpin et al., 2019), leading blue whales to forage in areas outside the GSL (Lesage et al., 2017). In the case of beluga, a ^13^C‐depletion was observed over time and may have resulted from a progressive shift of beluga distribution toward more ^13^C‐depleted habitat, such as the LE or the nwGSL in summer (Simard et al., 2023). Alternatively, beluga may have modified their diet to include a greater proportion of ^13^C‐depleted pelagic fishes (Lesage et al., 2020).

While the dataset exploited as part of this study is extensive, our study was subjected to sampling limitations, with some species having a small sample size and few individuals per year (i.e., between 6 and 10). The use of SEA_C_, which takes into account sample size, and of the relatively unbiased SEA_B_ overcome at least partly this issue (Jackson et al., 2011). Also, there might be a small bias in the sampling protocol for some of the species and years, where more than one individual from a same trawl tow were analyzed isotopically, potentially underestimating the overall variability of the isotopic space. The approach to analyze stable isotopes, to deal with lipid effects, and to preserve rorqual skin samples have changed over the course of the study. However, validation studies have been conducted to estimate and correct for each of these potential biases. For instance, isotopic values from samples preserved in DMSO can be reliably restored by the methodology explicitly developed by Lesage et al. (2010) for those samples (see details in Appendix S1). The conclusions of the current study have been shown to be similar when using food web structure issued from bulk samples or lipid‐free samples and thus, were deemed robust to the change in methodology for dealing with lipid effects (see details in Appendix S1). Particular care was also taken in the current study with field sampling (i.e., location, season, specimen size, and sample size) and laboratory analyses for the most recent period to insure comparability of the collected data among periods. Other potential biases that are difficult to account for include the possibility that rorquals or other migratory fish or invertebrate species are advected into the sampled area, or have migrated from other parts of the Gulf or the Estuary, or areas outside the EGSL prior to sampling. It is also possible that the timing of arrival of the rorquals has changed over time, modifying to some extent their isotopic signatures. The primary productivity and the phytoplankton and zooplankton community composition and abundance in the EGSL have changed since the 1990s (Blais et al., 2021; Plourde et al., 2014; Sorochan et al., 2019). While these changes do not seem to have affected the isotopic composition of community components unidirectionally, they still may have affected, to an unknown extent, the carbon and nitrogen baselines of the St. Lawrence ecosystem.

## 5 CONCLUSIONS

This study, based on a long‐term and substantial sampling effort (see e.g., Cabrol et al., 2021; Lesage et al., 2001; Ouellet et al., 2023), documented the evolution of the isotopic signatures and niches of 21 species of marine invertebrates, fishes, and mammals over time and space, and provides the isotopic baseline required for assessing predator diets. The observed variations suggest that collecting both predator and potential prey concomitantly from the same location and time period is essential to avoid complications when interpreting the trophic interactions and foraging ecology of predators. Furthermore, these results underscore the importance of a regular sampling of a set of sentinel species of ecosystems representing various trophic levels or carbon sources, including DIC and DIN, which may facilitate the interpretation of observed trends in species foraging ecology. The addition of complementary techniques, such as amino acid compound‐specific isotopic analysis (CSIA) or other isotopes, might help distinguishing effects from the baseline from those associated with predator diet in future analyses (Richards et al., 2020). Species distribution, abundance, and community assemblages are affected by climate change and anthropogenic stressors, with a likely continuing change in their ecological niche space and overlap. The degree of diet and niche plasticity, availability of unoccupied niches, and abundance of resources (Pansu et al., 2022) could be key to population persistence in the face of a changing environment.

## AUTHOR CONTRIBUTIONS

**Ève Rioux:** Conceptualization (lead); data curation (lead); formal analysis (lead); investigation (lead); methodology (lead); visualization (lead); writing – original draft (lead); writing – review and editing (lead). **Jory Cabrol:** Conceptualization (equal); data curation (equal); writing – review and editing (equal). **Véronique Lesage:** Conceptualization (equal); data curation (equal); funding acquisition (lead); project administration (lead); resources (lead); writing – review and editing (equal).

## Supporting information


Appendix S1.

Appendix S2.

## Data Availability

The data that supports the findings of this study are available in Appendix S2 of this article.
