# Supplementary material for: Long‐term evolution of the structure of the St. Lawrence (Canada) marine ecosystem in the context of climate change and anthropogenic activities: An isotopic perceptive
Source: Ecol Evol. 2023 Nov 28;13(11):e10740. doi: 10.1002/ece3.10740 (PMC10684986; doi:10.1002/ece3.10740)
Supplement: Supplementary file 1 — Appendix S1. [file ECE3-13-e10740-s001.doc]

**SUPPLEMENTARY MATERIAL**

**Long-term evolution of the structure of St. Lawrence (Canada) marine ecosystem in the context of climate change and anthropogenic activities: an isotopic perceptive**

Ève Rioux, Jory Cabrol, & Véronique Lesage

**Preservative and lipid-extraction corrections**

Isotopic values are predictably affected by dimethyl sulfoxide (hereafter referred to as DMSO) preservation and can be reliably restored by lipid extraction preceded by water rising, and by applying linear mathematical corrections explicitly developed by Lesage et al. (2010) for rorqual skins. The δ^13^C and δ^15^N values of rorqual skin samples preserved in DMSO (*n* = 189) were retro-corrected with the following equations:

δ^13^C_lipid-free_ = 0.911 * δ^13^C_lipid-free DMSO_ – 1.548

δ^15^N_bulk_ = 0.960 * δ^15^N_lipid-free DMSO_ + 0.371

Some samples from 1995 to 2003 (*n* = 553) were analyzed for isotope ratios from the same lipid-extracted aliquot. To account for lipid extraction effects on δ^15^N_bulk_ values, mathematical linear corrections were developed in a companion study by Ouellet et al. (2023) using the muscle tissue of several krill, fish, and marine mammal species, and are applied as follows:

δ^15^N_bulk_ = β_1_ * δ^15^N_lipid-free_ + β_0_ (eqn 1)

where β_0_ is the intercept and β_1_ is the slope. To improve the accuracy and precision of the retro-correction of δ^15^N_bulk_ values, Ouellet et al. (2023) developed a new approach based on clusterization, where resembling species were identified using mixture models (R package flexMix, Grün & Leisch, 2007, 2008; Leisch, 2004), based on similarities among species in the relationship of δ^15^N_bulk_ against δ^15^N_lipid-free_. The optimal number of clusters (k) was determined using the stepFlexMix function (R package flexMix) and Bayesian information criterion after repeated trials with candidate values of k ranging from 1 (all species combined) to each species considered separately (20 species). The optimal number of clusters of resembling species within our dataset was six. Posterior probabilities (i.e., g.prob, the probability for a species to be adequately assigned to a cluster, indicative of the robustness of the clusterization output) were high, ranging from 0.92 to 1.00 with the exception of Northern krill (i.e., 0.77). The intercept β_0_ and slope β_1_ were calculated using the mixture model (Table S1) and equation 1 (above) for each cluster, and the δ^15^N_bulk_ values were then retro-corrected using these specific β_0_ and β_1_ parameters.

In the absence of such correction factors for copepod species, a general equation for krill and fish species was applied given the similarity in the carbon to nitrogen elemental ratio (hereafter referred to as CN) between krill and fishes (CN = 3.1 ± 0.1), and copepods (CN = 3.3 ± 0.1). δ^15^N_bulk_ values of copepods were thus retro-corrected with a general coefficient estimated from equation 6 in Ouellet et al. (2023):

δ^15^N_bulk_ = 1.03 * δ^15^N_lipid-free_ – 1.05.

The approach to analyze stable isotope signatures and deal with lipid effect has changed over last decades. To ensure that the results obtained in this study were not due to the different laboratory procedures between periods, the SITA analyses (Sturbois et al., 2021) were repeated using δ^13^C_lipid-free_ and δ^15^N_lipid-free_. No differences were noted if we used δ^13^C_lipid-free_ and δ^15^N_lipid-free_ or δ^13^C_lipid-free_ and δ^15^N_bulk_. We were thus confident that differences observed between periods were not due to the laboratory procedures and show a real change in the community structure of the EGSL.

**Geographical location, species, and period effects on δ^13^C and δ^15^N values**

The effect of species, regions, periods, and their interactions on isotopic signatures were examined separately for both δ^13^C and δ^15^N using generalized linear models (GLMs) with a Gaussian distribution on a subset of data (i.e., Atlantic cod, capelin, Atlantic herring, American plaice, and American sand lance, *n* = 480). Since the species present in the three regions were not the same, only species present in the three regions were selected for this analysis. Models explaining variation in δ^13^C and δ^15^N signatures were highly significant (both *p* < 0.001), and the total variance explained by fixed effects was relatively high for δ^13^C (*R*^2^ = 0.57) and for δ^15^N (*R*^2^ = 0.80). The interaction between species, region, and period was also highly significant for both isotopes (*F*_5,455_ = 19.8; *p* < 0.001 for δ^13^C and *F*_5,455_ = 16.1; *p* < 0.001 for δ^15^N) and confirmed the need to consider region, species, and period in subsequent analyses.

**Ontogenic effect on δ^13^C and δ^15^N values**

Nitrogen isotope enrichment is documented with increasing body size within and across fish species due to the fact that they may shift their diet to prey at a higher trophic level, while a carbon enrichment may reflect habitat shifts and associated changes in diet (Cocheret de la Morinière et al., 2003; Jennings et al., 2002; Lesage et al., 2001). The effect of body size (i.e., total length) on δ^13^C and δ^15^N values was examined for 12 fish species by using linear regressions of isotopic values and total length of the organism. We assessed the normality of residuals using normal quantile–quantile plots and assessed heteroscedasticity by plotting residuals versus fitted values (Quinn & Keough, 2002; Zuur et al., 2010). If the assumptions were not met or a non-linear pattern was observed in residuals, a generalized additive model (GAM) was used instead of a linear regression and the most parsimonious model was selected based on the Akaike information criterion (AIC). GAMs were used to identify nonlinear trends with total length as the explanatory variable and cubic regression spline as the smoothing function. The “*gam.check*” function from the mgcv package (Pedersen et al., 2019) was used in order to determine if any of the fitted models adequately described patterns in the data and to determine if the number of degrees of freedom was adequate.

Isotopic signatures varied with total length in several fish species (Tables S2, S3), with an enrichment noted with body length in both δ^13^C (i.e., American plaice, Atlantic tomcod, capelin, Greenland halibut, rainbow smelt, and American sand lance) and δ^15^N values (i.e., American eel, American plaice, Arctic cod, Atlantic herring, Atlantic mackerel, Atlantic tomcod, Greenland halibut, rainbow smelt, and American sand lance). Atlantic cod and Atlantic mackerel were exceptions, as they showed a significant depletion in ^13^C with an increase in body length. Therefore, length classes for a given species were homogenized between periods for comparison. The representation of the size distribution was verified with a density plot using the ggplot 2 package (Wickham, 2016). All fish species were similar in size distribution between periods, with the exception of American eel and Atlantic mackerel: total length for American eel was on average 88.0 cm [71.5–104.0, min‒max] for 1995–2003 and 67.5 cm [58.0–77.1] for 2019–2021; total length for Atlantic mackerel was on average 27.2 cm [24.0–30.0, min‒max] for 1995–2003 and 11.4 cm [8.0–16.5] for 2019–2021. Mackerel were removed from the analysis because the small average in 2019–2021 could be associated with juveniles instead of adults. The majority of the northern contingent of Atlantic mackerel reach sexual maturity at around 2 and 3 years, and length at first maturity (L_50_) is estimated at 266 ± 1.5 mm (mean ± *SD*) (Smith et al., 2021). The American eel was kept for the subsequent analyses due to the weak ontogeny effect on stable isotope signatures of nitrogen and no effect on carbon (Table S2).

Table S1. Species groups based on clusterization of mixture model with their posterior probabilities (g.prob) and coefficient estimates (β_0_ and β_1_) of the linear model (eqn 1) for retro-correction of δ^15^N_bulk_ values.

| **Species** | **Group** | **g.Prob** | **Intercept β_0_** | **Slope**  **β1** |
| --- | --- | --- | --- | --- |
| American eel | 3 | 0.92 | -0.03 | 0.97 |
| American plaice | 5 | 1.00 | 3.83 | 0.69 |
| American sand lance | 6 | 1.00 | -0.24 | 0.96 |
| Arctic cod | 6 | 1.00 | -0.24 | 0.96 |
| Arctic krill | 1 | 0.98 | -0.15 | 0.96 |
| Atlantic cod | 3 | 1.00 | -0.03 | 0.97 |
| Atlantic herring | 4 | 1.00 | 2.96 | 0.71 |
| Atlantic tomcod | 1 | 0.98 | -0.15 | 0.96 |
| beluga | 2 | 1.00 | 0.70 | 0.95 |
| capelin | 4 | 1.00 | 2.96 | 0.71 |
| Greenland halibut | 4 | 1.00 | 2.96 | 0.71 |
| Northern krill | 3 | 0.77 | -0.03 | 0.97 |
| rainbow smelt | 1 | 0.98 | -0.15 | 0.96 |
| redfish | 6 | 1.00 | -0.24 | 0.96 |
| shrimps | 4 | 1.00 | 2.96 | 0.71 |

Table S2. Results of linear regressions of δ^13^C and δ^15^N as a function of total fish length. Significant regressions (*p* value < 0.05) are indicated in bold. The total length average and ranges are presented for each fish species; only specimens within the indicated size range were used in this study.

| Species | Total length (cm)  *x̄* [min-max] | *n* |  | δ^13^C | | |  | | δ^15^N | | | |  |
| --- | --- | --- | --- | --- | --- | --- | --- | --- | --- | --- | --- | --- | --- |
|  |  |  |  | *F* | *p* value | *R*^2^_adj_ | |  | | *F* | *p* value | *R*^2^_adj_ | |
| American eel | 77.5 [58.0—104.0] | 37 |  | 0.2 | 0.67 | -0.02 | |  | | **4.4** | **0.04** | **0.09** | |
| American plaice | 26.7 [13.4—41.0] | 62 |  | **25.0** | **< 0.001** | **0.28** | |  | | **24.2** | **< 0.001** | **0.28** | |
| American sand lance | 12.0 [8.3—15.5] | 51 |  |  |  |  | |  | | **7.9** | **0.01** | **0.12** | |
| Arctic cod | 13.4 [10.5—17.6] | 19 |  | 0.1 | 0.80 | -0.05 | |  | | **5.1** | **0.04** | **0.19** | |
| Atlantic cod | 31.8 [23.0—47.5] | 52 |  | **13.2** | **< 0.001** | **0.19** | |  | | 0.02 | 0.88 | -0.02 | |
| Atlantic herring | 24.3 [14.5—32.9] | 126 |  | 0.1 | 0.80 | -0.01 | |  | |  |  |  | |
| Atlantic mackerel | 16.0 [8.0—30.0] | 17 |  | **32.8** | **< 0.001** | **0.67** | |  | | **25.8** | **< 0.001** | **0.61** | |
| Atlantic tomcod | 22.1 [14.5—33.0] | 47 |  | **6.9** | **0.01** | **0.11** | |  | |  |  |  | |
| capelin | 13.9 [10.2—17.3] | 150 |  |  |  |  | |  | | 0.4 | 0.51 | -0.004 | |
| Greenland halibut | 30.2 [21.9—37.6] | 34 |  | **4.9** | **0.03** | **0.11** | |  | |  |  |  | |
| rainbow smelt | 16.8 [14.7—22.5] | 32 |  |  |  |  | |  | | **4.9** | **0.03** | **0.11** | |
| redfish | 25.0 [21.5—30.5] | 26 |  | 0.01 | 0.91 | -0.04 | |  | | 2.3 | 0.14 | 0.05 | |

Table S3. Results of generalized additive models of δ^13^C and δ^15^N as a function of total fish length. edf is the effective degrees of freedom, F is the F value, *p* is the *p* value of the model, and DE (%) is the percent of deviance (or variability) explained by the model. Significant models (*p* value < 0.05) are indicated in bold.

| Species | *n* |  |  | δ^13^C | | |  | |  | | δ^15^N | | | |
| --- | --- | --- | --- | --- | --- | --- | --- | --- | --- | --- | --- | --- | --- | --- |
|  |  |  | edf | *F* | *p* value | DE (%) | |  | | edf | | *F* | *p* value | DE (%) |
| American sand lance | 51 |  | **2.9** | **13.7** | **< 0.001** | **51.8** | |  | |  | |  |  |  |
| Atlantic herring | 126 |  |  |  |  |  | |  | | **3.6** | | **3.8** | **0.005** | **13.9** |
| Atlantic tomcod | 47 |  |  |  |  |  | |  | | **4.0** | | **6.8** | **< 0.001** | **47.1** |
| capelin | 150 |  | **3.5** | **2.6** | **0.03** | **8.9** | |  | |  | |  |  |  |
| Greenland halibut | 34 |  |  |  |  |  | |  | | **2.4** | | **7.1** | **0.001** | **42.1** |
| rainbow smelt | 32 |  | **2.5** | **3.5** | **0.03** | **31.1** | |  | |  | |  |  |  |

Table S4. Sample sizes (*n*), δ^13^C and δ^15^N values (mean ± *SE*), and trajectory metrics (net changes and angle α) for the various species sampled in the northwestern Gulf of St. Lawrence (nwGSL), and the Lower (LE) and Upper (UE) St. Lawrence Estuary over two periods (1995–2003 vs. 2019–2021). Latin names and body length (in cm) are indicated after species common names. The blue color indicates a depletion in δ^13^C or δ^15^N in recent period compared to the past period, while the orange color indicates an enrichment in δ^13^C or δ^15^N in the recent period.

| **Species** | **1995**–**2003** | | |  | **2019**–**2021** | | |  | **Trajectory metrics** | |
| --- | --- | --- | --- | --- | --- | --- | --- | --- | --- | --- |
|  | ***n*** | **δ^13^C** | **δ^15^N** |  | ***n*** | **δ^13^C** | **δ^15^N** |  | **Net changes** | **Angle α** |
| **nwGSL** |  |  |  |  |  |  |  |  |  |  |
| American plaice *Hippoglossoides platessoides* (13.4—41.0) | 10 | -18.9 ± 0.2 | 14.1 ± 0.1 |  | 20 | -18.9 ± 0.2 | 13.3 ± 0.1 |  | 0.9 | 182.8 |
| American sand lance *Ammodytes* sp. (8.3—15.5) | 17 | -19.1 ± 0.1 | 10.7 ± 0.1 |  | 6 | -21.1 ± 0.2 | 9.9 ± 0.2 |  | 2.1 | 248.4 |
| Arctic cod *Boreogadus saida* (10.5—17.6) | 9 | -20.1 ± 0.2 | 13.2 ± 0.4 |  | 10 | -19.2 ± 0.0 | 12.5 ± 0.1 |  | 1.1 | 129.0 |
| Atlantic cod *Gadus morhua* (25.1—46.6) | 7 | -20.0 ± 0.1 | 14.7 ± 0.2 |  | 30 | -19.0 ± 0.1 | 14.1 ± 0.1 |  | 1.2 | 122.1 |
| Atlantic herring *Clupea harengus* (16.3—30.3) | 10 | -20.3 ± 0.3 | 12.1 ± 0.2 |  | 29 | -19.5 ± 0.1 | 12.6 ± 0.1 |  | 1.0 | 56.2 |
| blue whale *Balaenoptera musculus* | 44 | -18.9 ± 0.1 | 10.1 ± 0.2 |  | 35 | -17.5 ± 0.1 | 10.2 ± 0.2 |  | 1.3 | 87.9 |
| capelin *Mallotus villosus* (10.2—16.3) | 21 | -19.1 ± 0.1 | 12.4 ± 0.1 |  | 38 | -19.5 ± 0.1 | 12.2 ± 0.1 |  | 0.5 | 244.4 |
| copepods *Calanus finmarchicus, C. hyperboreus, Metridia*  *longa, Paraeuchaeta norvegica* | 10 | -19.3 ± 0.1 | 9.2 ± 0.1 |  | 6 | -20.6 ± 0.3 | 9.3 ± 0.1 |  | 1.3 | 270.3 |
| fin whale *Balaenoptera physalus* | 78 | -18.9 ± 0.0 | 10.6 ± 0.2 |  | 19 | -18.9 ± 0.1 | 12.1 ± 0.2 |  | 1.5 | 357.0 |
| Greenland halibut *Reinhardtius hippoglossoides* (21.9—37.6) | 10 | -19.5 ± 0.1 | 13.6 ± 0.2 |  | 40 | -19.1 ± 0.1 | 12.7 ± 0.1 |  | 1.0 | 149.4 |
| humpback whale *Megaptera novaeangliae* | 62 | -18.9 ± 0.0 | 13.4 ± 0.1 |  | 23 | -19.1 ± 0.1 | 13.9 ± 0.2 |  | 0.6 | 335.3 |
| minke whale *Balaenoptera acutorostrata* | 10 | -18.9 ± 0.1 | 12.2 ± 0.6 |  | 9 | -18.7 ± 0.1 | 12.9 ± 0.3 |  | 0.7 | 14.1 |
| redfish *Sebastes* spp. (21.5—30.5) | 5 | -19.5 ± 0.1 | 13.4 ± 0.1 |  | 40 | -19.2 ± 0.0 | 12.2 ± 0.2 |  | 1.2 | 166.5 |
| **LE** |  |  |  |  |  |  |  |  |  |  |
| American plaice *Hippoglossoides platessoides* (18.4—39.6) | 6 | -18.6 ± 0.1 | 13.7 ± 0.3 |  | 30 | -18.1 ± 0.1 | 13.6 ± 0.1 |  | 0.5 | 97.4 |
| American sand lance *Ammodytes* sp. (9.6—15.4) | 14 | -19.4 ± 0.2 | 10.8 ± 0.1 |  | 15 | -19.3 ± 0.1 | 11.1 ± 0.1 |  | 0.3 | 13.6 |
| Arctic krill *Thysanoessa* spp. | 20 | -19.1 ± 0.1 | 9.3 ± 0.1 |  | 16 | -17.9 ± 0.1 | 9.2 ± 0.1 |  | 1.2 | 94.6 |
| Atlantic cod *Gadus morhua* (23.0—47.5) | 6 | -19.0 ± 0.3 | 13.4 ± 0.5 |  | 22 | -18.5 ± 0.1 | 14.2 ± 0.2 |  | 0.9 | 30.9 |
| Atlantic herring *Clupea harengus* (18.5—32.9) | 26 | -19.1 ± 0.2 | 12.6 ± 0.1 |  | 26 | -19.8 ± 0.1 | 12.4 ± 0.1 |  | 0.7 | 251.5 |
| beluga *Delphinapterus leucas* | 55 | -17.2 ± 0.0 | 16.0 ± 0.1 |  | 14 | -17.8 ± 0.1 | 16.1 ± 0.3 |  | 0.5 | 278.8 |
| capelin *Mallotus villosus* (11.5—17.3) | 43 | -19.5 ± 0.1 | 12.4 ± 0.1 |  | 33 | -19.2 ± 0.1 | 12.5 ± 0.1 |  | 0.3 | 72.3 |
| Northern krill *Meganyctiphanes norvegica* | 40 | -20.0 ± 0.1 | 10.6 ± 0.1 |  | 18 | -19.2 ± 0.2 | 10.4 ± 0.1 |  | 0.8 | 101.0 |
| shrimps *Pandalus borealis, P. montagui* (9.3—16.9) | 7 | -16.7 ± 0.1 | 10.8 ± 0.3 |  | 41 | -17.4 ± 0.1 | 12.2 ± 0.1 |  | 1.6 | 331.3 |
| **UE** |  |  |  |  |  |  |  |  |  |  |
| American eel *Anguilla rostrata* (58.0—104.0) | 18 | -22.0 ± 0.7 | 13.9 ± 0.4 |  | 19 | -21.9 ± 0.7 | 13.0 ± 0.2 |  | 0.9 | 176.9 |
| Atlantic herring *Clupea harengus* (14.5—25.4) | 22 | -18.1 ± 0.1 | 12.5 ± 0.1 |  | 15 | -19.3 ± 0.1 | 12.8 ± 0.8 |  | 1.2 | 284.3 |
| Atlantic tomcod *Microgadus tomcod* (14.5—33.0) | 21 | -18.7 ± 0.2 | 13.4 ± 0.3 |  | 26 | -18.0 ± 0.2 | 15.5 ± 0.1 |  | 2.2 | 19.0 |
| capelin *Mallotus villosus* (11.7—15.5) | 20 | -17.8 ± 0.3 | 13.2 ± 0.2 |  | 15 | -19.3 ± 0.6 | 12.0 ± 0.1 |  | 1.9 | 233.5 |
| rainbow smelt *Osmerus mordax* (14.7—22.5) | 7 | -19.7 ± 0.3 | 14.6 ± 0.3 |  | 25 | -19.3 ± 0.3 | 14.7 ± 0.1 |  | 0.4 | 82.5 |
| striped bass *Morone saxatilis* (18.7—25.3) | - | - | - |  | 24 | -17.8 ± 0.3 | 14.3 ± 0.1 |  | - | - |

Table S5. Differences (expressed in delta notation) in δ^13^C and δ^15^N values between periods (2019–2021 vs. 1995–2003) for the various species in the northwestern Gulf of St. Lawrence (nwGSL), and the Lower (LE) and Upper (UE) St. Lawrence Estuary. Significant effects are indicated in bold. The blue color indicates a significant depletion in δ^13^C or δ^15^N in the recent period compared to the past period, while the orange color indicates a significant enrichment in δ^13^C or δ^15^N in the recent period.

|  | **Δ^13^C (‰)** | **Δ^15^N (‰)** |
| --- | --- | --- |
| **nwGSL** |  |  |
| American plaice | 0.0 ± 0.3 | **-0.9 ± 0.6** |
| American sand lance | **-2.0 ± 0.4** | -0.8 ± 0.8 |
| Arctic cod | **0.8 ± 0.4** | -0.7 ± 0.8 |
| Atlantic cod | **1.0 ± 0.4** | -0.6 ± 0.7 |
| Atlantic herring | **0.8 ± 0.3** | 0.5 ± 0.6 |
| blue whale | **1.3 ± 0.2** | 0.1 ± 0.4 |
| capelin | **-0.4 ± 0.2** | -0.2 ± 0.5 |
| copepods | **-1.3 ± 0.5** | 0.0 ± 0.9 |
| fin whale | -0.1 ± 0.2 | **1.5 ± 0.4** |
| Greenland halibut | **0.5 ± 0.3** | -0.8 ± 0.6 |
| humpback whale | -0.2 ± 0.2 | **0.5 ± 0.4** |
| minke whale | 0.2 ± 0.4 | 0.7 ± 0.8 |
| redfish | 0.3 ± 0.4 | **-1.2 ± 0.8** |
| **LE** |  |  |
| American plaice | 0.5 ± 0.4 | -0.1 ± 0.6 |
| American sand lance | 0.1 ± 0.4 | 0.3 ± 0.5 |
| Arctic krill | **1.2 ± 0.3** | -0.1 ± 0.4 |
| Atlantic cod | 0.5 ± 0.4 | 0.8 ± 0.6 |
| Atlantic herring | **-0.7 ± 0.3** | -0.2 ± 0.4 |
| beluga | **-0.5 ± 0.3** | 0.1 ± 0.4 |
| capelin | 0.3 ± 0.2 | 0.1 ± 0.3 |
| Northern krill | **0.8 ± 0.3** | -0.2 ± 0.3 |
| shrimps | **-0.8 ± 0.4** | **1.4 ± 0.5** |
| **UE** |  |  |
| American eel | 0.1 ± 1.0 | **-0.9 ± 0.6** |
| Atlantic herring | -1.2 ± 1.0 | 0.3 ± 0.6 |
| Atlantic tomcod | 0.7 ± 0.9 | **2.0 ± 0.5** |
| capelin | **-1.5 ± 1.0** | **-1.1 ± 0.6** |
| rainbow smelt | 0.4 ± 1.3 | 0.1 ± 0.8 |


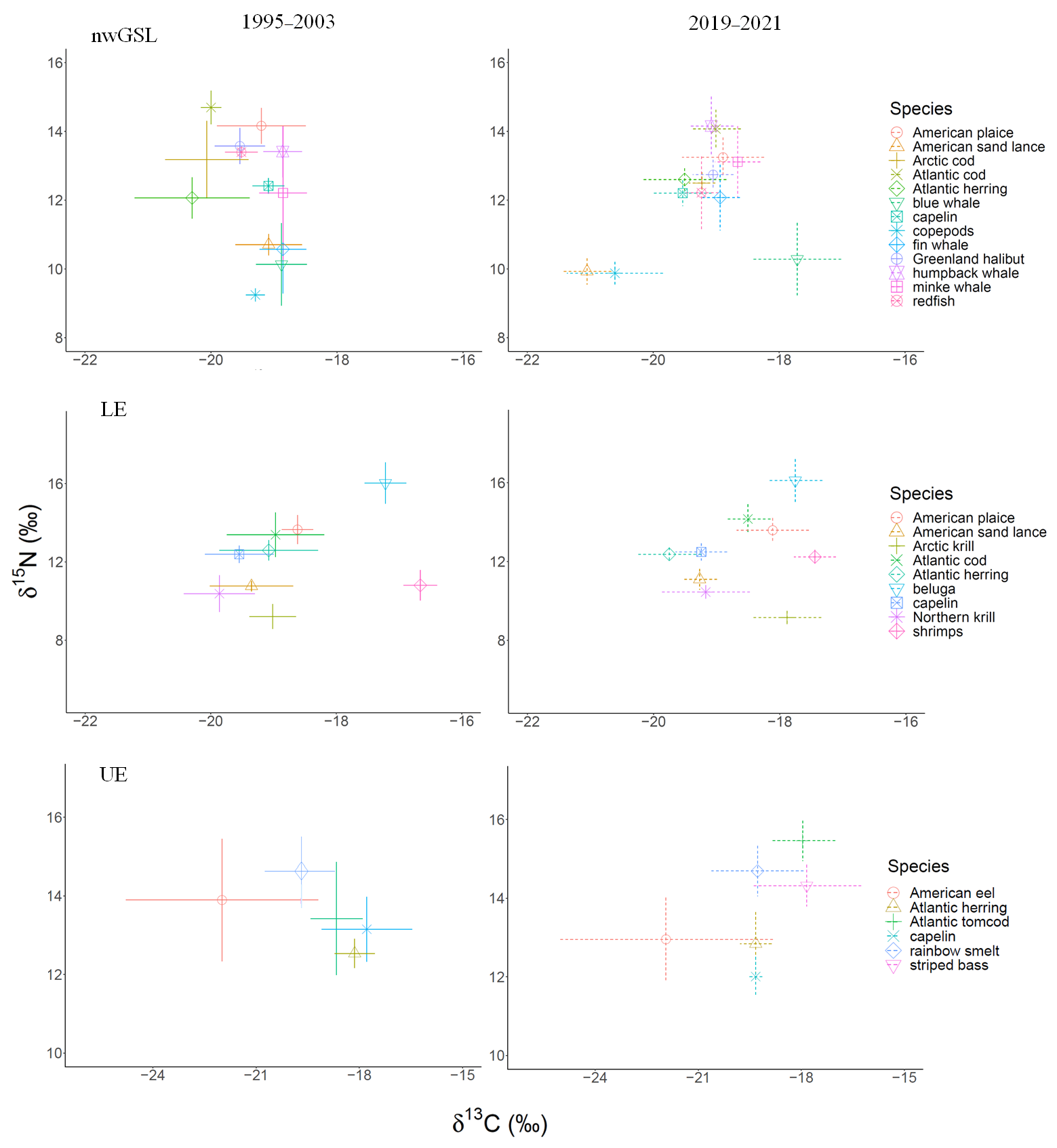


Figure S1. Mean stable isotope signature (δ^13^C and δ^15^N) with error bars (± *SD*) of various species sampled in the northwestern Gulf of St. Lawrence (nwGSL), and the Lower (LE) and Upper (UE) St. Lawrence Estuary over two periods (1995–2003 vs. 2019–2021).


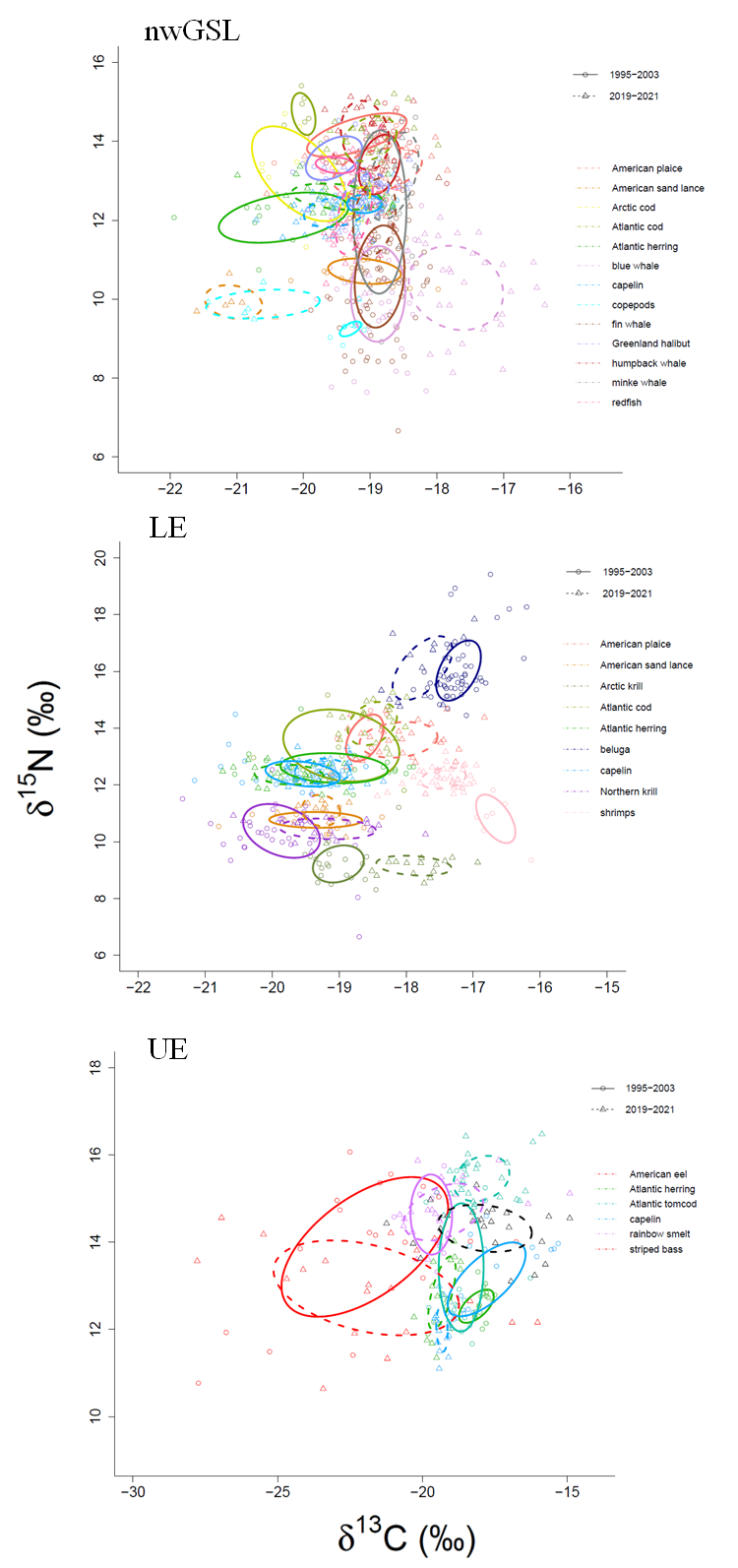


Figure S2. Bayesian standard ellipse areas (SEA_B_) in the bidimensional isotopic space of δ^13^C and δ^15^N values of various species for periods 1995–2003 (ο) and 2019–2021 (Δ), and for the northwestern Gulf of St. Lawrence (nwGSL), and the Lower (LE) and Upper (UE) Estuary regions. SEA_B_ contain 40% of the data. δ^13^C and δ^15^N values are plotted for each organism belonging to a species according to period.


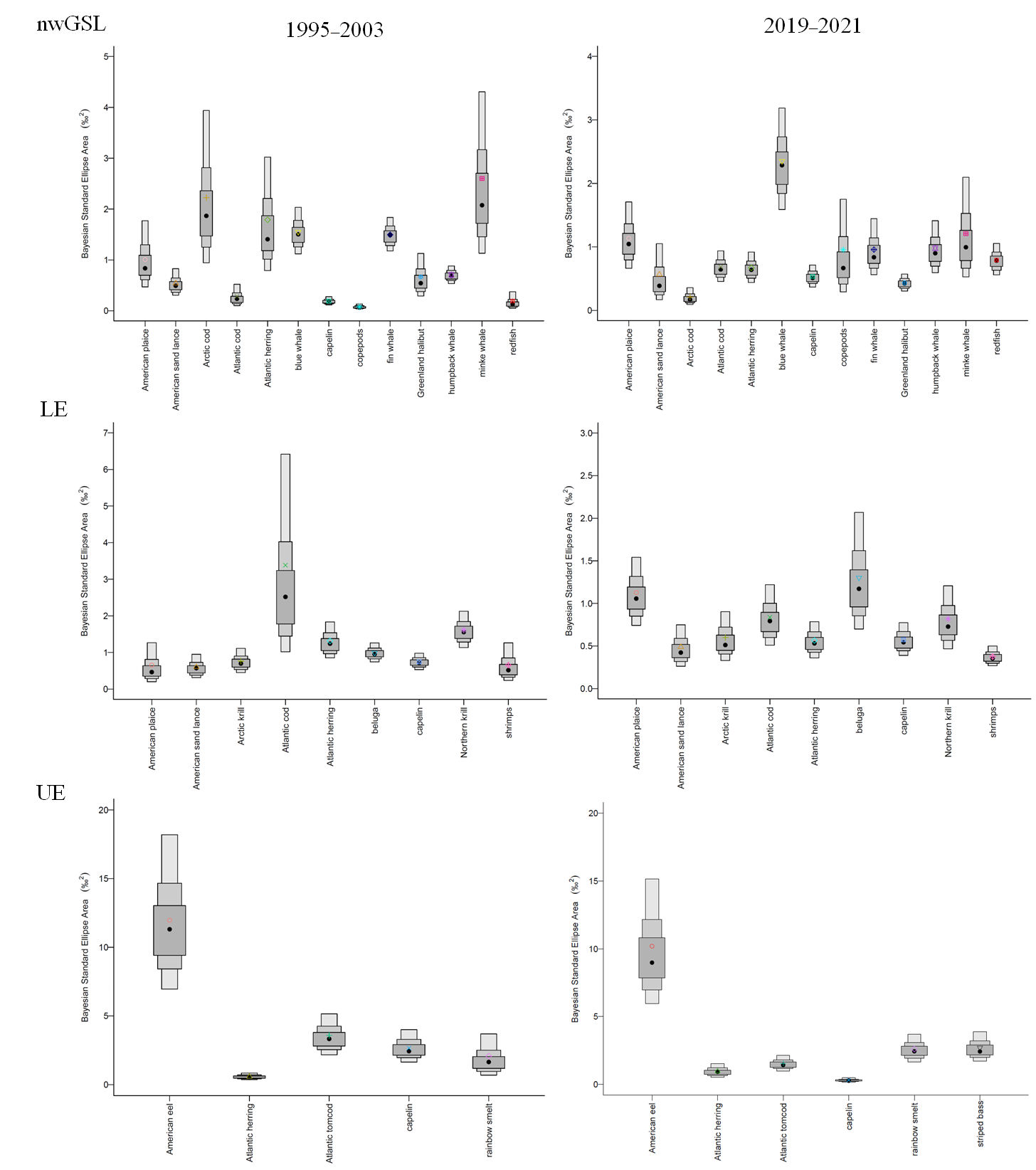


Figure S3. Bayesian standard ellipse areas (SEA_B_) and credible intervals 95% values of both periods (1995–2003 vs. 2019–2021) for the northwestern Gulf of St. Lawrence (nwGSL), and the Lower (LE) and Upper (UE) Estuary regions. The black dots represent the SEA_B_ mode, the colored dots represent the SEA_C_ mode, and the shaded boxes represent the 50%, 75% and 95% credible intervals from dark to light grey.

REFERENCES

Cocheret de la Morinière, E., Pollux, B. J. A., Nagelkerken, I., Hemminga, M. A., Huiskes, A. H. L., & Van der Velde, G. (2003). Ontogenetic dietary changes of coral reef fishes in the mangrove-seagrass-reef continuum: Stable isotopes and gut-content analysis. *Marine Ecology Progress Series*, *246*, 279–289. https://doi.org/10.3354/meps246279

Grün, B., & Leisch, F. (2007). Fitting finite mixtures of generalized linear regressions in R. *Computational Statistics & Data Analysis*, *51*(11), 5247–5252.

Grün, B., & Leisch, F. (2008). FlexMix version 2: Finite mixtures with concomitant variables and varying and constant parameters. *Journal of Statistical Software*, *28*(4), 1–35.

Jennings, S., Greenstreet, S. P. R., Hill, L., Piet, G. J., Pinnegar, J. K., & Warr, K. J. (2002). Long-term trends in the trophic structure of the north sea fish community: Evidence from stable-isotope analysis, size-spectra and community metrics. *Marine Biology*, *141*(6), 1085–1097. https://doi.org/10.1007/s00227-002-0905-7

Leisch, F. (2004). FlexMix: A general framework for finite mixture models and latent class regression in R. *Journal of Statistical Software*, *11*(8), 1–18.

Lesage, V., Hammill, M. O., & Kovacs, K. M. (2001). Marine mammals and the community structure of the Estuary and Gulf of St Lawrence, Canada: Evidence from stable isotope analysis. *Marine Ecology Progress Series*, *210*, 203–221. https://doi.org/10.3354/meps210203

Lesage, V., Morin, Y., Rioux, È., Pomerleau, C., Ferguson, S. H., & Pelletier, É. (2010). Stable isotopes and trace elements as indicators of diet and habitat use in cetaceans: predicting errors related to preservation, lipid extraction, and lipid normalization. *Marine Ecology Progress Series*, *419*, 249–265. https://doi.org/10.3354/meps08825

Ouellet, J.-F., Cabrol, J., Rioux, È., Bordeleau, X., & Lesage, V. (2023). Dealing with lipid effects and lipid-extraction biases in isotopic studies: a solution based on 28 marine invertebrate, fish and mammal species. b*ioRxiv*. https://doi.org/10.1101/2023.10.25.563823.

Pedersen, E. J., Miller, D. L., Simpson, G. L., & Ross, N. (2019). Hierarchical generalized additive models in ecology: An introduction with mgcv. *PeerJ*, *2019*(5). https://doi.org/10.7717/peerj.6876

Quinn, G. P., & Keough, M. J. (2002). *Experimental design and data analysis for biologists*. Cambridge University Press. https://doi.org/10.1016/S0022-0981(02)00278-2

Smith, A., Girard, L., Boudreau, M., Van Beveren, E., & Plourde, S. (2021). Assessment of the northern contingent of Atlantic mackerel (*Scomber scombrus*) in 2020*. DFO Can. Sci. Advis. Sec. Sci. Advis. Rep. 2021/029*. iv + 38 p.

Sturbois, A., Cucherousset, J., De Cáceres, M., Desroy, N., Riera, P., Carpentier, A., Quillien, N., Grall, J., Espinasse, B., Cherel, Y., & Schaal, G. (2021). Stable Isotope Trajectory Analysis (SITA): A new approach to quantify and visualize dynamics in stable isotope studies. *Ecological Monographs*, e1501. https://doi.org/10.1002/ecm.1501

Wickham, H. (2016). *ggplot2: Elegant graphics for data analysis.* Springer-Verlag.

Zuur, A. F., Ieno, E. N., & Elphick, C. S. (2010). A protocol for data exploration to avoid common statistical problems. *Methods in Ecology and Evolution*, *1*, 3–14. https://doi.org/10.1111/j.2041-210x.2009.00001.x
